# Identification of a novel endogenous long non-coding RNA that inhibits selenoprotein P translation

**DOI:** 10.1093/nar/gkab498

**Published:** 2021-06-18

**Authors:** Yuichiro Mita, Risa Uchida, Sayuri Yasuhara, Kohei Kishi, Takayuki Hoshi, Yoshitaka Matsuo, Tadashi Yokooji, Yoshino Shirakawa, Takashi Toyama, Yasuomi Urano, Toshifumi Inada, Noriko Noguchi, Yoshiro Saito

**Affiliations:** The Systems Life Sciences laboratory, Department of Medical Life Systems, Faculty of Life and Medical Sciences, Doshisha University, Kyotanabe 610-0394, Japan; The Systems Life Sciences laboratory, Department of Medical Life Systems, Faculty of Life and Medical Sciences, Doshisha University, Kyotanabe 610-0394, Japan; The Systems Life Sciences laboratory, Department of Medical Life Systems, Faculty of Life and Medical Sciences, Doshisha University, Kyotanabe 610-0394, Japan; The Systems Life Sciences laboratory, Department of Medical Life Systems, Faculty of Life and Medical Sciences, Doshisha University, Kyotanabe 610-0394, Japan; Laboratory of Molecular Biology and Metabolism, Graduate School of Pharmaceutical Sciences, Tohoku University, Aoba-ku, Sendai 980-8578, Japan; Laboratory of Gene Regulation, Graduate School of Pharmaceutical Sciences, Tohoku University, Sendai 980-8578, Japan; The Systems Life Sciences laboratory, Department of Medical Life Systems, Faculty of Life and Medical Sciences, Doshisha University, Kyotanabe 610-0394, Japan; The Systems Life Sciences laboratory, Department of Medical Life Systems, Faculty of Life and Medical Sciences, Doshisha University, Kyotanabe 610-0394, Japan; Laboratory of Molecular Biology and Metabolism, Graduate School of Pharmaceutical Sciences, Tohoku University, Aoba-ku, Sendai 980-8578, Japan; The Systems Life Sciences laboratory, Department of Medical Life Systems, Faculty of Life and Medical Sciences, Doshisha University, Kyotanabe 610-0394, Japan; Laboratory of Gene Regulation, Graduate School of Pharmaceutical Sciences, Tohoku University, Sendai 980-8578, Japan; The Systems Life Sciences laboratory, Department of Medical Life Systems, Faculty of Life and Medical Sciences, Doshisha University, Kyotanabe 610-0394, Japan; The Systems Life Sciences laboratory, Department of Medical Life Systems, Faculty of Life and Medical Sciences, Doshisha University, Kyotanabe 610-0394, Japan; Laboratory of Molecular Biology and Metabolism, Graduate School of Pharmaceutical Sciences, Tohoku University, Aoba-ku, Sendai 980-8578, Japan

## Abstract

Selenoprotein P (SELENOP) is a major plasma selenoprotein that contains 10 Sec residues, which is encoded by the UGA stop codon. The mRNA for SELENOP has the unique property of containing two Sec insertion sequence (SECIS) elements, which is located in the 3′ untranslated region (3′UTR). Here, we coincidentally identified a novel gene, *CCDC152*, by sequence analysis. This gene was located in the antisense region of the SELENOP gene, including the 3′UTR region in the genome. We demonstrated that this novel gene functioned as a long non-coding RNA (lncRNA) that decreased SELENOP protein levels via translational rather than transcriptional, regulation. We found that the CCDC152 RNA interacted specifically and directly with the SELENOP mRNA and inhibited its binding to the SECIS-binding protein 2, resulting in the decrease of ribosome binding. We termed this novel gene product lncRNA inhibitor of SELENOP translation (L-IST). Finally, we found that epigallocatechin gallate upregulated *L-IST* in vitro and in vivo, to suppress SELENOP protein levels. Here, we provide a new regulatory mechanism of SELENOP translation by an endogenous long antisense ncRNA.

## Graphical Abstract

**Graphical Abstract:**
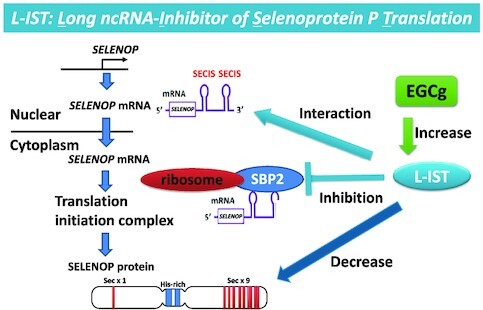
L-IST directly interacts with SELENOP mRNA, inhibits the binding of SECIS-binding protein 2 (SBP2) and ribosome to SELENOP mRNA, and finally decreases SELENOP protein expression. Epigallocatechin gallate (EGCg) upregulated L-IST to suppress SELENOP protein levels.

## INTRODUCTION

Selenium (Se), which is an essential trace element, is mainly incorporated into proteins as a selenocysteine (Sec) residue (an analogue of cysteine containing Se instead of sulphur) (1). Twenty-five types of Sec-containing proteins, i.e. selenoproteins, have been identified in humans. These proteins play an important role in several physiological processes: glutathione peroxidase (GPx) functions for the removal of several hydroperoxides, thioredoxin reductase (TrxR) for redox regulation, selenophosphate synthetase for Sec synthesis and iodothyronine deiodinases for the regulation of thyroid hormones (1–3). Thus, several dysfunctions, such as thyroid dysfunction, spermatogenesis dysfunction, and the increase of cancer risk, are induced by the deficiency of Se and selenoproteins (4–7).

Sec is encoded by the UGA codon, whose normal function is to terminate translation, and its translation involves a unique mechanism (8,9). In eukaryotes, the Sec insertion sequence (SECIS), which is a specific hairpin structure located in the 3′ untranslated region (3′UTR) of selenoprotein mRNA, is essential for the incorporation of Sec during the biosynthesis of selenoproteins (10,11). SECIS binds the SECIS-binding protein 2 (SBP2) and forms a complex for Sec translation via the recruitment of the eukaryotic elongation factor for Sec translation (eEFsec) and Sec-tRNA^[Ser]Sec^ (an anticodon complimentary to the UGA codon) (12). SBP2 is stably associated with ribosomes and contains a distinct L7Ae RNA-binding domain, which binds the SECIS element with high affinity and specificity (13). The eEFSec binds to both SBP2 and Sec-tRNA^[Ser]Sec^. When the ribosome and the canonical translation complex reach an UGA codon, Sec is inserted from the SECIS complex and premature termination is prevented. In addition to these factors, other factors, such as the eukaryotic initiation factor 4a3 and the ribosomal protein L30 (RPL30), are related to the translation of Sec (14).

Selenoprotein P (SELENOP), which is encoded by *SELENOP*, is a major selenoprotein in the plasma that is mainly produced in the liver (15,16). SELENOP is the only selenoprotein that contains multiple Sec residues that are important for its function. One N-terminal Sec residue forms an active site of GPx-like enzyme activity to reduce phospholipid hydroperoxide, while the nine C-terminal Sec residue function as a Se transporter to deliver Se to the cells effectively (17–19). The mRNA of SELENOP has the unique property of containing two SECIS elements, as other selenoprotein mRNAs have only one SECIS element in their 3′UTR (20,21). It has been reported that the first SECIS, which is located on the 5′ side near the stop codon, mainly facilitates the processive Sec incorporation, while the second SECIS functions slow decoding at the first UGA codon (22). However, the mechanisms of Sec translation in SELENOP and the regulation of the expression of SELENOP are not fully understood.

More than 98% of the genome does not code for mRNAs, and the biological role of the non-coding genomic sequence, such as intergenic regions, has been receiving much attention. In recent years, many nuclear and cytoplasmic non-coding RNAs (ncRNAs) have been reported (23,24). Some of these nuclear ncRNAs can regulate protein levels, e.g. nuclear ncRNAs control the epigenetic state of the promoter regions of genes, participate in transcriptional regulation, are involved in alternative splicing, constitute subnuclear compartments and export nuclear mRNAs (25–27). Recent studies have reported that some types of antisense cytoplasmic ncRNAs, such as miRNA/siRNA (28), Linc RNA p21 (29) and the SINEB2 repeat-containing RNA (30), regulate mRNA levels. Furthermore, a fraction of sense ncRNAs, such as competing endogenous RNAs (ceRNAs), enhance the levels of target mRNAs by trapping and inhibiting miRNAs (31,32).

In the present study, we coincidentally identified a novel gene by sequence analysis. This gene is located in the antisense region of the SELENOP gene in the genome. We investigated the function of this novel gene and found that it suppresses SELENOP translation. Here, we provide a new regulatory mechanism of SELENOP translation by an endogenous long antisense ncRNA.

## MATERIALS AND METHODS

### Cell culture

Human hepatoma Li-7 were purchased from Cell Resource Center for Biomedical Research in Tohoku University (Sendai, Japan). Other all cell lines used here were obtained from the American Type Culture Collection (Manassas VA, USA). Human embryonic kidney (HEK293) cells and human hepatocellular carcinoma (HepG2) cells were maintained in Dulbecco's modified Eagle's medium (DMEM; Gibco Thermo Fisher Scientific, Waltham MA, USA) containing 10% heat-inactivated foetal bovine serum (FBS) and antibiotics (100 U/ml penicillin and 100 μg/ml streptomycin; Invitrogen, Thermo Fisher Scientific). Neuroblastoma SH-SY5Y cells were maintained in DMEM/F12 medium (Gibco) containing 10% FBS and antibiotics. Malignant glioma U-87 MG cells were maintained in MEM (Gibco) containing 10% FBS and antibiotics. Human T-cell lymphocyte Jurkat cells were maintained in RPMI 1640 medium (Sigma-Aldrich, St. Louis, MO, USA) containing 10% FBS and antibiotics. All cell lines were cultured at 37°C under an atmosphere of 95% air and 5% CO_2_. FBS and EGCg (E4143) was purchased from Sigma-Aldrich. All other chemicals used in this work were of the highest quality commercially available.

### Animal experiments

All animal experiments described in this study were approved by the Animal Care Committee of Doshisha University (approval no. A16035) and conformed fully to the guidelines outlined in the Guide for the Care and Use of Laboratory Animals of Japan. Eight-weeks-old male C57BL/6J mice were obtained from Shimizu Laboratory Supplies (Kyoto, Japan). All animals were housed in a 12/12 h light/dark cycle and had free access to food and water. Blood glucose levels were determined by the glucose oxidase method (Glutest Sensor; Sanwa Kagaku, Kyoto, Japan).

### Real-time PCR analysis

Total RNA was extracted from cells and tissues using the TriPure Isolation Reagent (Roche, Mannheim, Germany) and reverse transcribed using a PrimeScript RT reagent kit (Takara, Shiga, Japan). Quantitative real-time reverse transcription PCR was performed using the power SYBR Green PCR Master Mix (Thermo Fisher Scientific) and a 7900HT Fast Real Time PCR System (Applied Biosystems, Foster City, CA, USA), according to the manufacturer's suggestions. The expression levels of target genes were normalized to the expression levels of RLP32 or 18S rRNA. The sequences of the primers used are provided in Supplementary Table S1.

### Plasmids and transfection

Human SELENOP with a SECIS sequence was a gift from Dr. Misu (Kanazawa University) and was re-inserted into the BamHI site of pcDNA3.1 (Thermo Fisher Scientific) using an In-Fusion HD Cloning Kit (Thermo Fisher Scientific). The human CCDC152 gene was amplified via PCR from U-87 MG cell line cDNA. The human CCDC152 gene, its short variants, and the antisense sequence of the SECIS region of selenoproteins were inserted into the BamHI site of pcDNA3.1 (Thermo Fisher Scientific) or pcDNA 4 (Thermo Fisher Scientific) using an In-Fusion HD Cloning Kit. The HA tag was inserted by the site-directed mutagenesis method. Plasmids were transfected using Lipofectamine LTX (Thermo Fisher Scientific), according to the manufacturer's instructions. Human CCDC152 RNAs were transiently transfected using Lipofectamine RNAiMAX (Thermo Fisher Scientific), according to the manufacturer's instructions.

### Western blotting

To obtain whole-cell lysates, treated cells were suspended in lysis buffer (50 mM Tris–HCl pH 7.5, 150 mM NaCl, 1% NP40, 0.1% SDS, 1% sodium deoxycholic acid with a cocktail of protease inhibitors (Nacalai Tesque, Kyoto, Japan) and phosphatase inhibitors (PhosSTOP; Roche) at 4°C for 30 min. Nuclei and unlysed cellular debris were removed by centrifugation at 15 000 × *g* for 5 min. The concentration of protein was determined using a Bicinchoninic Acid Protein Assay Kit (Pierce Biotechnology, Rockford, IL, USA) with bovine serum albumin as the standard. The protein samples were separated by sodium dodecyl sulphate-polyacrylamide gel electrophoresis (SDS-PAGE) and subjected to western blotting with appropriate antibodies. The antibodies used for immunoblotting included anti-SELENOP (33), anti-β-actin (1:10 000, A5441; Sigma), anti-GPx1 (1:1000, ab22607; Abcam, Cambridge, UK), anti-GPx4 (1:1000, ab125066; Abcam), anti-TrxR1 (34), anti-TrxR2 (1:1000, HPA003323; Sigma), anti-GAPDH (1:40 000, ab8245; Abcam), anti-lamin A/C (1:1000, #2032; Cell Signaling, Danvers, MA, USA) and anti-SBP2 (1:1000, 12798-1-AP; Protein Tech) antibodies. HRP-conjugated secondary antibodies were obtained from Santa Cruz Biotechnology (Santa Cruz, CA, USA) and Jackson Immuno Research (West Grove, PA, USA). The immunoreactivity in the PVDF membrane (Millipore, Billerica, MA, USA) was visualized using the Immobilon Western HRP Substrate (Millipore) and an LAS-4000 luminescence imager (Fujifilm, Tokyo, Japan).

### Reverse transcriptional PCR

Total RNA was extracted from cells using the TriPure Isolation Reagent (Roche, Mannheim, Germany) and reverse transcribed using a Revertra Ace (Toyobo, Osaka, Japan) with oligo dT (Invitrogen, Carlsbad, CA, USA) or random primers (Invitrogen, Carlsbad, CA, USA). Reverse transcriptional PCR was performed using the TaKaRa Ex Taq (Takara) and a TaKaRa PCR Thermal Cycler Dice® Touch (Takara), according to the manufacturer's suggestions.

### Subcellular fractionation of cells

Cells were suspended with 10 mM HEPES–KOH buffer (pH 8.0) containing 10 mM KCl, 1.5 mM MgCl_2_ and 1 mM DTT and homogenized by Dounce Tissue Grinder at 4°C. The cytosolic fractions were obtained from supernatant by centrifugation at 1300 × *g* for 5 min. The pellet was suspended with 10 mM Tris–HCl buffer (pH 7.9) containing 8.55% Sucrose, 5 mM MgCl_2_, 10 mM DTT, and 0.01% Triton X-100 and after centrifugation at 1300 × *g* for 5 min, the pellet was suspended with lysis buffer and used as the nuclear fraction. Protease inhibitors were added to each fraction. The distribution of each subcellular fraction was judged by standard proteins such as lamin A/C (nuclear marker) and GAPDH (cytosolic marker).

### *In vitro* RNA transcription and RNA antisense purification (RAP)

Each RNA was synthesized using a TranscriptAid T7 High Yield Transcription Kit (Thermo Fisher Scientific). We performed RAP as described previously (35,36), with modifications. The CCDC152 RNA was biotinylated using a Biotin RNA Labeling mix (Roche). We used 2 million HepG2 cells and 3 μg of biotin-labelled CCDC152 RNA per experiment, as well as incubation in a highly denaturing buffer (20 mM Tris–HCl [pH 7.5], 50 mM KCl, 1.5 mM MgCl_2_, 0.4% sodium deoxycholate [Sigma], 1% NP-40 [Sigma], 1% *N*-lauroylsarcosine [Sigma], 2 mM DTT [Sigma], 1% protease inhibitor cocktail [nacalai tesque] and 40 U/ml RNase inhibitor [Takara]) at 37°C for 2 h. RNAs that interacted with the biotin-labelled CCDC152 RNA were isolated from whole-cell extracts using avidin magnetic beads (Invitrogen, Carlsbad, CA, USA) and the TriPure isolation reagent (Roche) and were then subjected to reverse transcription using a PrimeScript RT reagent kit (Takara). Reverse transcription PCR was performed using Ex Taq (Takara).

### Polysome analysis

We performed a polysome analysis as described previously (37), with modifications. Polysome profiles were obtained using sucrose density gradients. HepG2 cells were transfected with pcDNA4 or *CCDC152* plasmid for 72 h before polysome analysis. HepG2 cells were harvested, and cell extracts were layered onto linear sucrose density gradients [10–50% sucrose in 20 mM HEPES–NaOH (pH 7.6), 100 mM KCl, 5 mM MgCl_2_, 1 mM dithiothreitol and 100 μg/ml cycloheximide] that had been prepared in open-top polyclear tubes (Seton Scientific, CA, USA) with the use of a Gradient Master. Samples were centrifuged at 284 000 × *g* for 80 min at 4°C in a P40ST rotor (Hitachi Koki, Tokyo, Japan). Gradients were then fractionated (Towa, Tsukuba, Japan). Polysome profiles were generated by continuous measurement of absorbance at 254 nm with a single-path UV-1 optical unit (AC-5200, ATTO, Tokyo, Japan) connected to a chart recorder (ATTO). Twenty fractions of equal volume were collected and RNA was extracted using the Isogen II (NIPPON GENE, Tokyo, Japan). RNA levels of each fraction were measured by NanoDrop (Thermo Scientific). RNA levels of each fraction were measured, and a fixed volume of each RNA sample was subjected to reverse transcription and qPCR. The ratio of RNA levels in each fraction was calculated as the relative Ct value to total RNA.

### RNA pull-down assay

We performed a SECIS and SBP2 interaction as described previously (38) with modifications. The SELENOP mRNA was synthesized using a TranscriptAid T7 High Yield Transcription Kit (Thermo Fisher Scientific). The SELENOP mRNA was biotinylated using a Biotin RNA Labeling mix (Roche). We used 2.5 million HepG2 cells and 2 μg of biotin-labelled SELENOP mRNA and GFP mRNA per experiment, as well as incubation in a pull-down buffer (50 mM HEPES [pH 7.5], 150 mM NaCl, 1.5 mM 0.5% Triton X-100, 1% protease inhibitor cocktail [nacalai tesque] and 40 U/ml RNase inhibitor [Takara]) at 4°C for overnight. The proteins that interacted with the biotin-labelled SELENOP mRNA were isolated from whole-cell extracts using avidin magnetic beads (Invitrogen, Carlsbad, CA, USA).

### Transfection of small interfering RNA

The human *CCDC152*-small interfering RNAs (siRNA) were designed and manufactured by Thermo Fisher Scientific, according to the current guidelines for effective knockdown by this method, respectively. The target sequences for h*CCDC152*-siRNA (Invitrogen, 5′-ACAAGGAGAUUGCAAUUCUUCGUAA-3′ [forward] and 5′-UUACGAAGAAUUGCAAUCUCCUUGU-3′ [reverse]) were used. The siRNAs were transfected into HepG2 cells by Lipofectamine RNAi MAX (Thermo Fisher Scientific). After transfection, cells were treated with EGCg and subjected to western blotting and real-time PCR analysis.

### Statistical analysis

Data are shown as the mean ± standard deviation (SD, *in vitro*) or standard error of mean (SEM, *in vivo*). Statistical comparisons were performed using the two-tailed unpaired Student's *t* test or analysis of variance (ANOVA) by running post hoc tests (for data involving more than two groups). A probability value (*P* value) < 0.05 was considered indicative of statistical significance. Statistical analyses were performed using Excel (Microsoft, Redmond, WA, USA) and SPSS software (IBM SPSS, Armonk, NY, USA).

## RESULTS

### Identification of a novel gene with sequences that are complementary to selenoprotein P

**Figure 1.**
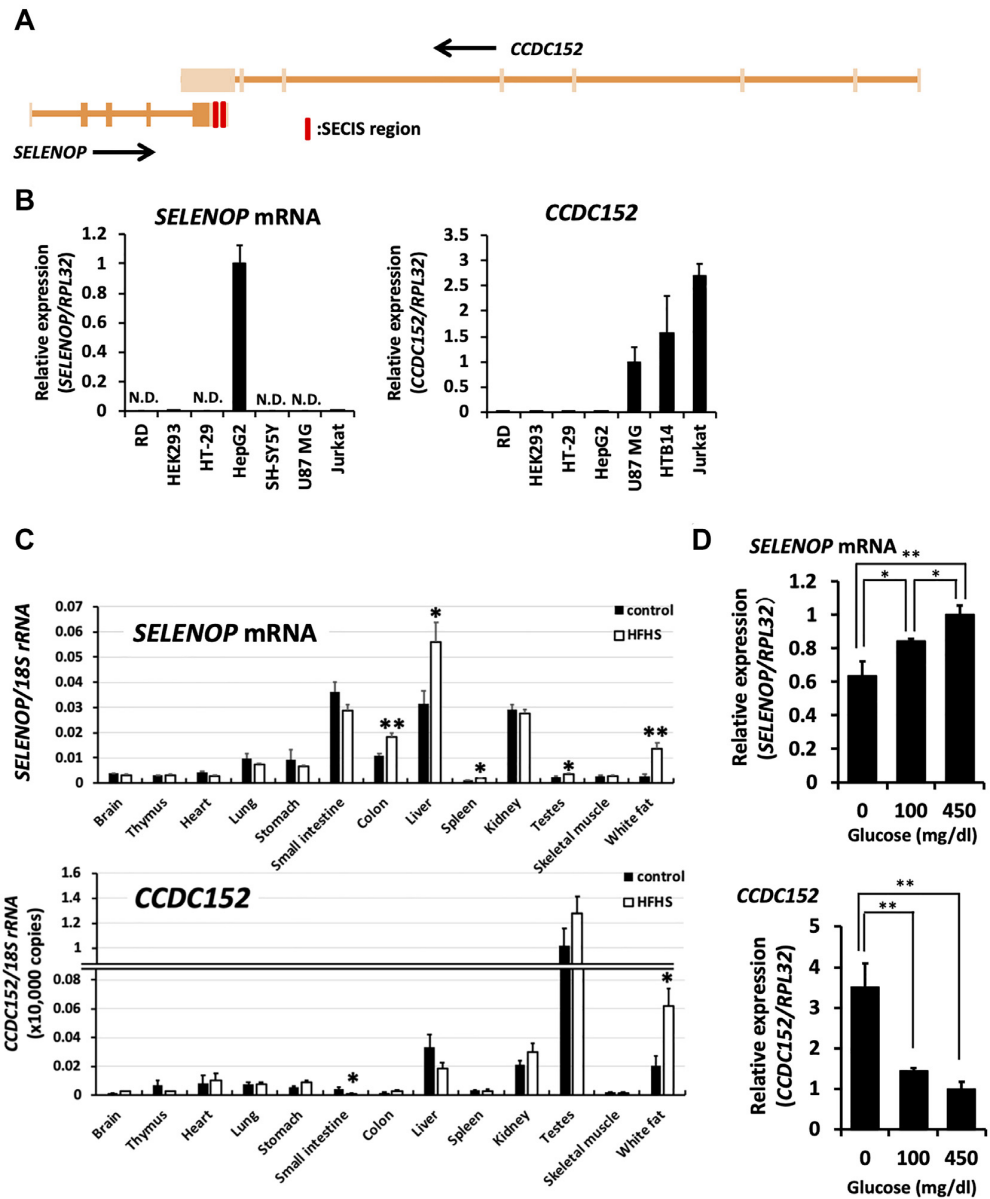
Expression of the CCDC152 gene in cells and tissues. (**A**) Schematic diagram of the sequence of SELENOP and the CCDC152 gene. The exons of each gene are shown in bold. The SECIS region is indicated as a red bar. (**B**) Relative expression levels of SELENOP and of the CCDC152 gene in each cell line. Each cell was harvested for RNA isolation and then analysed by real-time PCR. The expression levels of the human selenoprotein P (SELENOP, left panel) and human CCDC152 (right panel) RNAs were normalized to that of the RPL32 mRNA (*n* = 3, mean ± SD). N.D.: not detectable. (**C**) Relative expression levels of SELENOP and CCDC152 gene in each tissue of mice that has been fed a high-fat, high-sucrose diet (HFHSD). RNA isolated from each mouse tissue from animals who were fed a normal diet or HFHSD for 16 weeks was subjected to real-time PCR analysis (*n* = 5, mean ± SEM) **P < 0.01, *P < 0.05 versus Control, Student's *t* test. (**D**) Effects of glucose concentration on the expression levels of *SELENOP* and *CCDC152*. HepG2 cells were treated with the indicated concentrations of glucose for 24 h, harvested for RNA isolation, and then analysed by real-time PCR. The expression levels of *SELENOP* (upper panel) and *CCDC152* (lower panel) were normalized to that of the RPL32 mRNA (*n* = 3, mean ± SD). **P < 0.01, *P < 0.05, Tukey-ANOVA.

We obtained the sequence of the SECIS element of SELENOP gene from SelenoDB (http://selenodb.crg.eu) and then conducted a Basic Local Alignment Search Tool (BLAST) analysis of this SECIS element on Nucleotide collection databases. As a result, we found a gene called the coiled coil domain-containing protein 152 (CCDC152) gene, which is located in the antisense region of the SELENOP gene in the genome (Figure 1A). The CCDC152 gene exhibited a complete antisense sequence, including the SECIS element of SELENOP mRNA. Next, we compared the gene expression levels of SELENOP and CCDC152. The SELENOP mRNA was mostly expressed in human hepatoma HepG2 cells, whereas the PCR products of the SELENOP gene, although low, were detected in human embryonic kidney 293 cells (HEK293 cells) and T lymphoma Jurkat cells, but not detectable in other human cell lines in the experimental condition used (Figure 1B). On the other hand, CCDC152 gene expression was mostly observed in human neuroblastoma SH-SY5Y cells, glioma U-87MG cells and Jurkat cells. The PCR products of the CCDC152 gene were low but detected in other cell lines including rhabdomyosarcoma RD cells, HEK293 cells, and human colorectal adenocarcinoma HT-29 cells (Figure 1B). The expression levels of the SELENOP and CCDC152 genes were determined in mouse organs. We found that the SELENOP mRNA was mostly expressed in the small intestine, liver, and kidney, while the CCDC152 gene was mostly expressed in the testis, followed by the liver, kidney, and white adipose tissue (Figure 1C and Supplementary Figure S1A). The increase in SELENOP expression in the presence of high levels of glucose has been documented; therefore, we investigated the effects of glucose on the expression of both the SELENOP and CCDC152 genes. We found that the expression of these genes was significantly altered by glucose concentration in HepG2 cells, namely the increase in the SELENOP gene expression by high glucose while the decrease in the CCDC152 gene expression (Figure 1D). The changes in SELENOP and CCDC152 gene expression levels were further evaluated in mice that were fed a high-fat, high-sucrose diet (HFHSD) for 16 weeks (which led to the increase in blood glucose and SELENOP levels). A significant increase in SELENOP expression was observed in the liver as well as the colon, spleen, testes and white adipose tissue of mice that were fed a HFHSD (Figure 1C). As for the CCDC152 gene, its expression levels in the small intestine and white adipose tissue were significantly changed, and its expression in the liver tended to be decreased in mice that were fed a HFHSD (Figure 1D). These results suggest that the gene expressions of SELENOP and CCDC152 are changed by glucose levels.

### Specific suppressive effect of the CCDC152 gene on SELENOP levels

**Figure 2.**
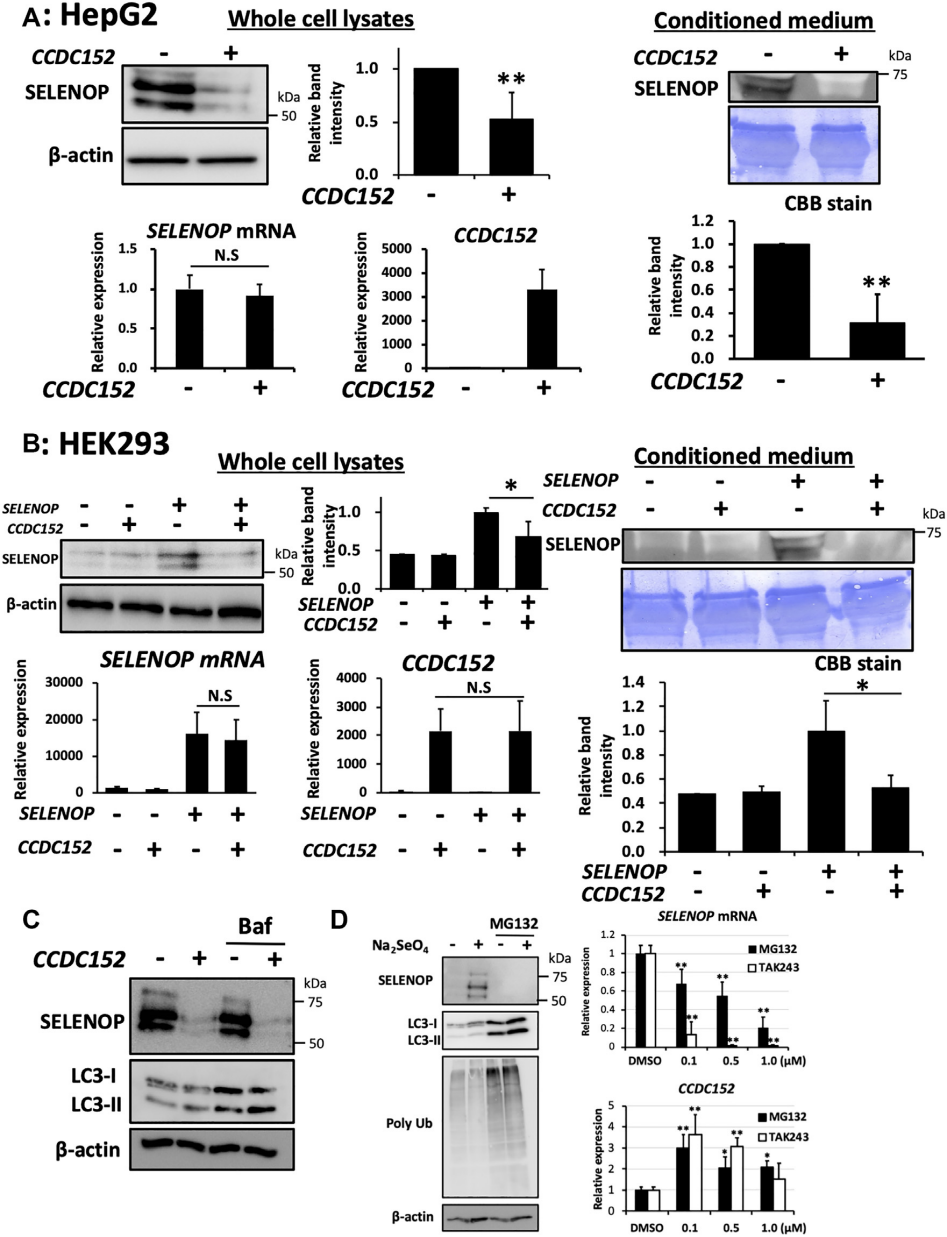
Suppressive effects of the CCDC152 gene on SELENOP levels. (**A**) Effects of CCDC152 gene expression on SELENOP levels in HepG2 cells. HepG2 cells were treated with *CCDC152* plasmid DNA or control plasmid DNA for 48 h, and then whole-cell lysates, extracted total RNA, and conditioned medium were subjected to western blotting with an anti-SELENOP Ab and real-time PCR, respectively (*n* = 3, mean ± SD). **P < 0.01 versus Control, Student's *t* test. (**B**) Effects of CCDC152 gene expression on SELENOP levels in HEK293 cells. HEK293 cells were treated with *CCDC152* plasmid DNA, *SELENOP* plasmid DNA, or control plasmid DNA in the presence or absence of 500 nM sodium selenite for 48 h, and then whole-cell lysates, extracted total RNA, and conditioned medium were subjected to western blotting and real-time PCR, respectively (*n* = 3, mean ± SD). *P < 0.05, Tukey-ANOVA. (**C**) Effects of bafilomycin A1 on the decrease of SELENOP levels induced by CCDC152 gene in HepG2 cells. HepG2 cells were treated with *CCDC152* plasmid DNA or control plasmid DNA for 48 h, and then whole cell lysates were subjected to western blotting. Bafilomycin A1 (5 nM) was added 24 h before the preparation of cell lysates. (**D**) Effects of MG132 and TAK243 on the SELENOP protein and the SELENOP and CCDC152 gene expression levels in HepG2 cells. HepG2 cells were treated with 1 μM MG132 in the presence or absence of 100 nM sodium selenite for 24 h, and then whole-cell lysates were subjected to western blotting. In the case of real-time PCR analysis, HepG2 cells were treated with indicated concentration of MG132 and TAK243 in the presence of 100 nM sodium selenite for 24 h (*n* = 3, mean ± SD). **P < 0.01 versus DMSO control, Tukey-ANOVA.

To further investigate the relationship between the SELENOP and CCDC152 genes, we examined the effects of *CCDC152* expression on the protein and mRNA levels of SELENOP. Transient expression of *CCDC152* in HepG2 cells [*SELENOP(+)/CCDC152(-)*] resulted in the statistically significant downregulation of SELENOP protein levels in both whole-cell lysates and conditioned medium, while the endogenous SELENOP mRNA levels were not changed (Figure 2A). In the case of human hepatoma Li-7 cells, the decrease in SELENOP protein levels in whole-cell lysates was also induced by *CCDC152* transfection without the significant change of SELENOP mRNA levels (Supplementary Figure S1B). In the case of HEK293 cells [*SELENOP(-)/CCDC152(-)*], SELENOP protein levels in both whole-cell lysates and conditioned medium were significantly decreased by co-expression with *SELENOP* and *CCDC152* (Figure 2B). Comparing the results of Figure 2A and B, it was considered that *CCDC152* transfection may show a suppressing effect only when the expression level of SELENOP was high. *CCDC152* overexpression did not affect the protein and mRNA levels of other selenoproteins, such as GPx1, GPx4, TrxR1 and TrxR2, which are Sec-containing proteins with a SECIS element, in HepG2 and HEK293 cells (Supplementary Figure S2A and B). These results suggest that the effect of the CCDC152 gene is specific to SELENOP protein expression, and not to other selenoproteins.

We next examined the effects of protease inhibitors to evaluate the involvement of protein degradation on the decrease of SELENOP protein levels induced by *CCDC152*. The addition of bafilomycin A1, a specific inhibitor of the vacuolar type H^+^-ATPase to prevent autophagosome–lysosome fusion, resulted in the increase of LC3-II, while bafilomycin A1 did not affect the decrease of SELENOP protein levels induced by *CCDC152* (Figure 2C). We also examined the effect of MG132, a cell-permeable proteasome and calpain inhibitor, on SELENOP protein levels. The sole treatment of MG132 resulted in the increase of LC3-II and polyubiquitinated proteins (Figure 2D). We found that MG132 treatment significantly decreased SELENOP protein levels in HepG2 cells in the presence of sodium selenite (Figure 2D). Further, the decrease in SELENOP mRNA levels and the increase in the CCDC152 gene were observed by the treatment with MG132 in the presence of sodium selenite (Figure 2D). The changes of these expression levels were also observed by the treatment with TAK-243, an inhibitor of the ubiquitin-activating enzyme, with unknown mechanisms (Figure 2D). It is interesting to note that both inhibitors significantly increased CCDC152 gene expression levels (Figure 2D). These results of proteasome inhibitors include unexpected effects that needed to be clarified with further research. Collectively, these results suggest that at least, the decrease of SELENOP protein levels induced by *CCDC152* did not involve the proteasomal degradation. Collectively, these results suggest that the CCDC152 gene specifically decreases SELENOP protein levels independent of protein degradation.

### The nature of the product of *CCDC152* and analysis of its sequence that is essential for the downregulation of SELENOP

**Figure 3.**
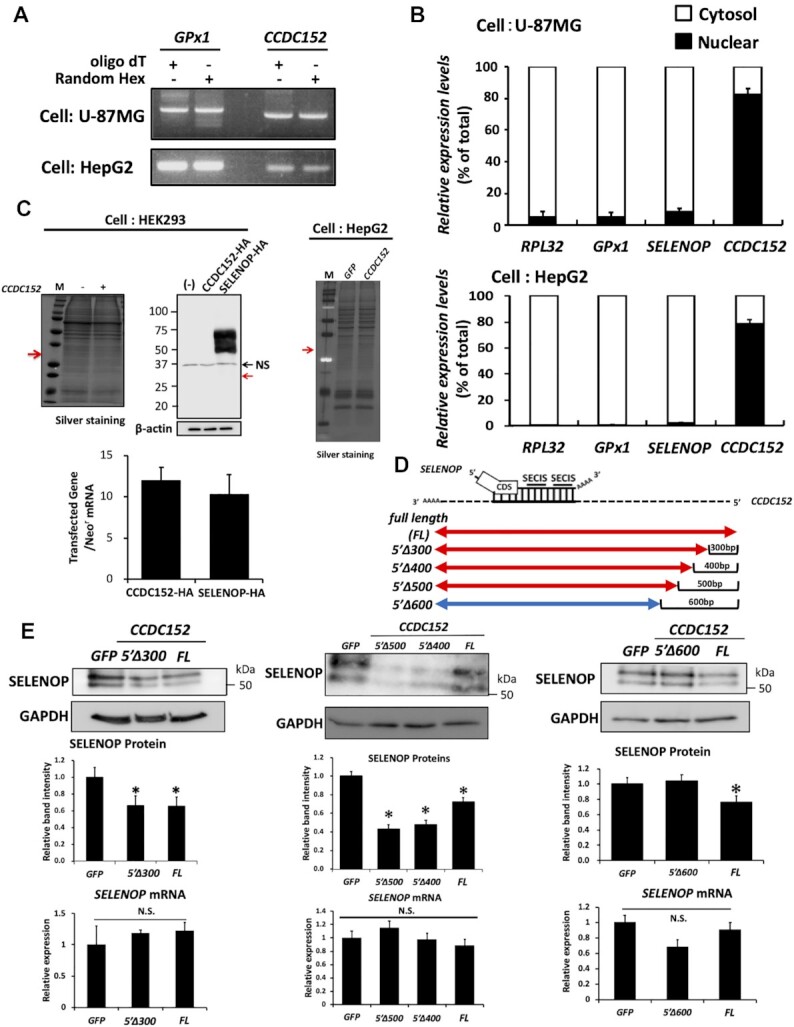
The nature of the product of *CCDC152* and its sequence that is essential to suppress SELENOP levels. (**A**) PCR analysis using an oligo dT primer and random hexamers. U-87MG cells and HepG2 cells were harvested for RNA isolation, and then real-time PCR was performed using an oligo dT primer and random hexamers. The PCR product was electrophoresed and visualized for the analysis. (**B**) Analysis of the cellular distribution of the product of CCDC152 gene. Nuclear and cytoplasmic fractions of U-87MG cells and HepG2 cells were prepared. The RNA in each fraction was separated and subjected to real-time PCR analysis for CCDC152 (*n* = 3, mean ± SD). (**C**) Effects of CCDC152 gene overexpression on cellular protein levels. HEK293 cells and HepG2 cells were treated with *CCDC152* plasmid DNA or *SELENOP* plasmid DNA with HA-tag or control GFP plasmid DNA for 48 h, and then extracted proteins were separated by SDS-PAGE and visualized using silver staining and western blotting with an anti-HA Ab. The expected molecular weight of the CCDC152 protein is indicated by a red arrow. After transfection, total RNA was also subjected to real-time PCR analysis. The expression levels of each transfected gene (CCDC152-HA and SELENOP-HA) were normalized to that of the neomycin-resistant gene (Neo^r^) mRNA (*n* = 3, mean ± SD). (**D**, **E**) Effects of CCDC152 RNA fragments on SELENOP levels of HepG2 cells. The full-length CCDC152 RNA and its fragments were synthesized in vitro and were transfected into HepG2 cells. A synthesized GFP mRNA was used as a control. After transfection, whole-cell lysates and total RNA were subjected to western blotting and real-time PCR analysis, respectively (*n* = 3, mean ± SD). *P < 0.05 versus GFP control, Dunnett-ANOVA. 5′Δ300–600, deletion of 300–600 bp of the 5′ region.

Next, we characterized the nature of the CCDC152 gene. Based on the PCR products obtained using an oligo dT primer and random hexamers, this gene was considered to have a poly A tail (Figure 3A). A cellular distribution analysis suggested that the product of *CCDC152* is localized mainly in the nuclei of both U-87 MG cells and HepG2 cells, which expressed *CCDC152* endogenously (Figure 3B). *CCDC152* overexpression in HEK293 cells and HepG2 cells did not yield an obvious protein band (Figure 3C). Further, the protein band of *CCDC152* was not observed by western blot analysis using an antibody against HA-tag, while that of SELENOP with HA-tag was detected as a positive control (Figure 3C). The stability of transfected CCDC152 RNA with HA-tag was confirmed (Figure 3C). These results suggest the role of this gene as a long ncRNA (lnc RNA).

To evaluate the role of the CCDC152 gene as a lncRNA and identify the sequence of this gene that is essential to suppress SELENOP expression, we examined the effects of the following CCDC152 RNA fragments: full length (FL), overlapping region (OL), 3′UTR region (3′UTR), anti-SECIS region (a-S), deletion of the 5′ region without overlapping (Δ5′) and deletion of the 3′ region without overlapping (Δ3′) (Supplementary Figure S3A). Transfection of *FL-CCDC152* suppressed SELENOP levels in HepG2 cells, while the other fragments did not (Supplementary Figure S3B and C). The stability of transfected CCDC152 RNA fragments was confirmed (Supplementary Figure S3B). These results suggest an essential role for both the *OL* and other regions in *CCDC152* in the suppression of SELENOP levels. Further, we prepared the following CCDC152 RNA fragments deleted from 300 to 600 bp of the 5′ region (*5*′*Δ300–600*) (Figure 3D and E). As shown in Figure 3E, transfection of *FL-*, *5*′*Δ300-, 5*′*Δ400-* and *5*′*Δ500-CCDC152* suppressed SELENOP levels in HepG2 cells, while *5*′*Δ600-CCDC152* fragment did not. These results suggest the essential role of the sequence from 500 bp of the 5′ region to suppress SELENOP protein levels (Figure 3D and E).

### Direct interaction between the CCDC152 RNA and the SELENOP mRNA

**Figure 4.**
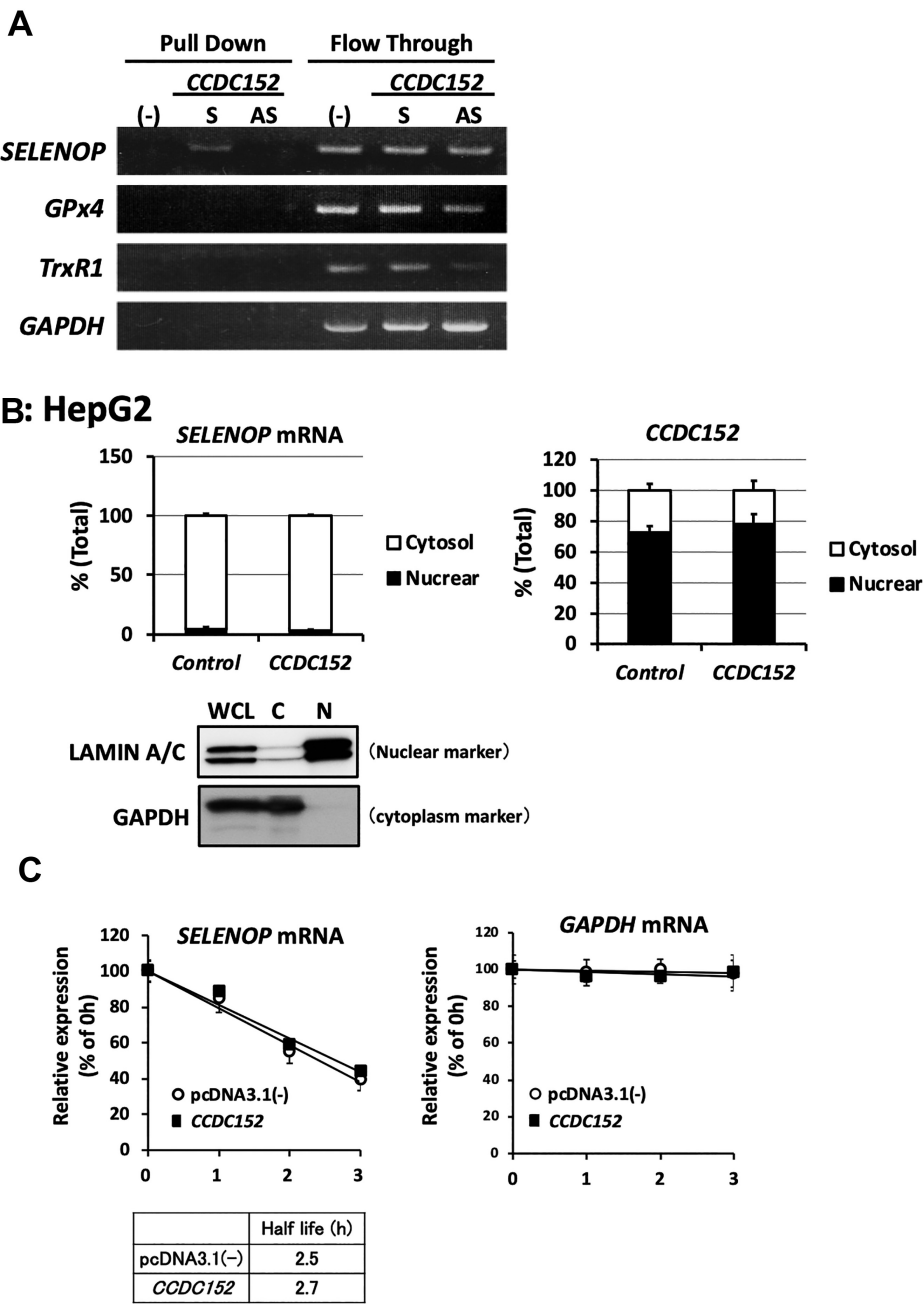
Direct interaction between the CCDC152 RNA and the SELENOP mRNA and effects of CCDC152 gene on the cellular distribution and degradation speed of the SELENOP mRNA. (**A**) Specific interaction between the CCDC152 RNA and the SELENOP mRNA. Biotinylated CCDC152 sense (S) and antisense (AS) RNAs were mixed with a synthesized mRNA, pulled down by avidin beads; subsequently, the precipitants and flow-through fractions were subjected to PCR analysis. (**B**) Effects of the CCDC152 gene on the cellular distribution of the SELENOP mRNA. The *CCDC152* and control plasmids were transfected into HepG2 cells and nuclear and cytoplasmic fractions were prepared. The quality of this fractionation was confirmed by the levels of lamin A/C (nuclear) and GAPDH (cytoplasm). RNA in each fraction was separated and subjected to real-time PCR analysis (*n* = 3, mean ± SD). (**C**) Effects of the CCDC152 gene on the degradation speed of the SELENOP mRNA. The *CCDC152* and control plasmids were transfected into HepG2 cells; in the presence of actinomycin D, SELENOP and GAPDH mRNA levels were determined at the indicated time points (*n* = 3, mean ± SD).

Next, we investigated the molecular mechanisms of SELENOP suppression induced by the CCDC152 gene. First, we investigated whether the interaction between the CCDC152 RNA and the SELENOP mRNA using a pull-down assay. A synthesized SELENOP mRNA and a biotinylated CCDC152 sense or antisense RNA were mixed and the precipitates were analysed by PCR. As shown in Figure 4A, co-precipitation of the SELENOP mRNA with the biotinylated sense CCDC152 RNA, but not with the antisense CCDC152 RNA, was observed. In addition, we found that the CCDC152 RNA did not interact with other SECIS-containing mRNAs and the GAPDH mRNA (Figure 4A), suggesting a specific direct interaction between the sense CCDC152 RNA and the SELENOP mRNA.

Subsequently, we examined the effects of the CCDC152 gene on SELENOP mRNA distribution, as a shift from the nucleus to the cytoplasm is necessary for translation. The cellular distribution of the CCDC152 RNA was compared with that of the SELENOP mRNA using nuclear and cytoplasmic fractions of *CCDC152*-plasmid-transfected HepG2 cells. In these cells, SELENOP mRNA derived from the endogenous promoter of the SELENOP gene was mainly distributed in the cytosolic fraction (Figure 4B). In contrast, the major CCDC152 RNA, which was overexpressed by a plasmid containing the cytomegalovirus promoter, was detected in the nuclear fraction. Based on the results shown in Figures 3B and 4B, it is thought that not only endogenous but also overexpressed CCDC152 RNA is mainly distributed in the nuclear fraction. As shown in Figure 4B, CCDC152 gene overexpression did not change the distribution of the SELENOP mRNA. We also found that the speed of degradation of the SELENOP mRNA in *CCDC152*-overexpressing HepG2 cells was not changed (Figure 4C). These results suggest that the CCDC152 RNA interacts with the SELENOP mRNA and that the CCDC152 gene does not affect the distribution or the degradation speed of the SELENOP mRNA.

### *CCDC152* inhibited the interaction between the SELENOP mRNA and SBP2

**Figure 5.**
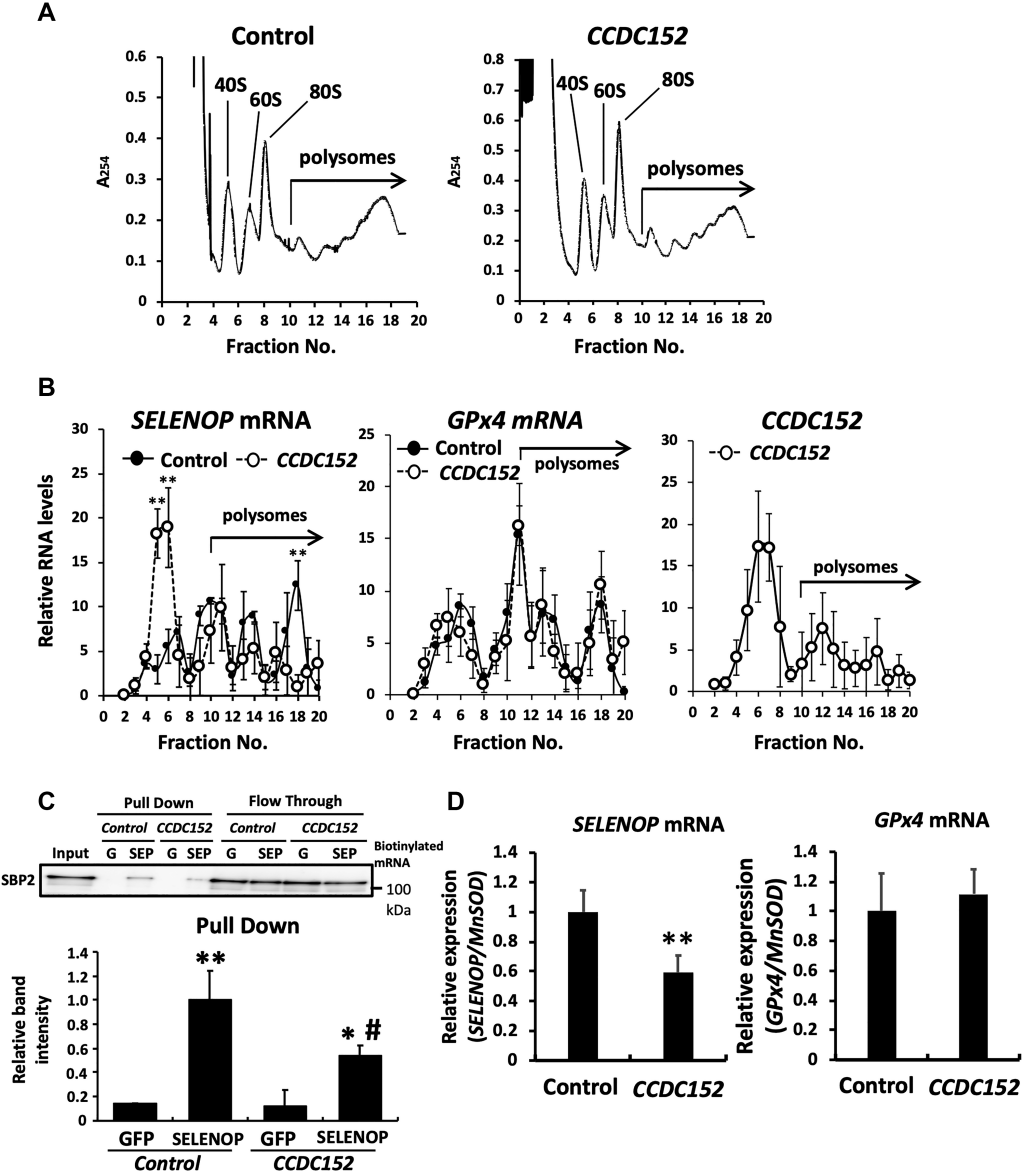
Effects of the CCDC152 gene on the interaction between the SELENOP mRNA and ribosomes and the SBP2 protein. (**A**, **B**) Effects of CCDC152 gene transfection on total and SELENOP translation in HepG2 cells. The control and *CCDC152* plasmids were transfected into HepG2 cells and the binding of ribosomes to total mRNA was determined by sucrose-gradient polysome analysis. Ribosome fractionation was revealed by measurement of absorbance at 254 nm (*A*_254_). Fractions 10 and 20 were assayed as polysomes (A). In the polysome analysis, the binding of ribosomes to the SELENOP mRNA, GPx4 mRNA, and CCDC152 RNA was determined. RNA in each fraction was extracted, its levels were measured and then a fixed volume of each RNA sample was subjected to quantitative PCR. The ratio of RNA levels in each fraction was calculated as the relative Ct value to total RNA (B, at least *n* = 3, mean ± SD). **P < 0.01 versus Control, Student's *t* test. (**C**, **D**) Effects of CCDC152 gene transfection on the interaction between the SELENOP mRNA and SBP2. The control and *CCDC152* plasmids were transfected into HepG2 cells and whole-cell lysates were prepared. Biotinylated-GFP mRNA and SELENOP mRNA was added to each cell lysate and the binding of the SBP2 protein to each mRNA was evaluated (C). The precipitants were subjected to western blotting using an anti-SBP2 antibody, and the band intensity of SBP2 was evaluated (*n* = 3, mean ± SD). **P < 0.01, *P < 0.05, versus GFP, #*P* < 0.05, versus SELENOP, control plasmid, Tukey-ANOVA. Anti-SBP2 Ab was added to each nuclear extracted fraction and the binding of the SELENOP and GPx4 mRNA to SBP2 was evaluated by the quantitative PCR (D). The MnSOD mRNA levels were used as a reference mRNA in the nuclear fraction (*n* = 3, mean ± SD). **P < 0.01 versus Control, Student's *t* test.

The SELENOP mRNA binds to ribosomes in the cytoplasm, which triggers translation. To analyse the effect of the CCDC152 gene on the interaction between the SELENOP mRNA and ribosomes, which reflects the translational level of proteins, we evaluated the binding of ribosomes to the SELENOP mRNA using a polysome analysis. We observed that there was no obvious change in polysome profiles between the control and *CCDC152*-transfected cells (Figure 5A). We also checked that CCDC152 overexpression did not influence cell growth and morphology (Supplementary Figure S4A-C). In turn, the SELENOP mRNA levels in fraction 18 of *CCDC152*-transfected HepG2 cells, where the SELENOP mRNA highly interacted with ribosomes, were significantly decreased, while the SELENOP mRNA with low ribosome binding in the lower fractions significantly increased (Figure 5B). These results suggest that the interaction between SELENOP mRNA and ribosomes was significantly decreased because of the overexpression of the CCDC152 gene (Figure 5B). Conversely, the GPx4 mRNA, which contains one SECIS in its mRNA, was not affected by *CCDC152* overexpression (Figure 5B). In addition, the interaction of CCDC152 RNA with ribosome was low (Figure 5B). These results suggest that the CCDC152 gene specifically inhibits the binding of ribosomes to the SELENOP mRNA.

In selenoproteins, the interaction between the SECIS element and SBP2 plays an important role in translation (39,40). To investigate the effect of the CCDC152 gene on the interaction between SBP2 and the SELENOP mRNA, we performed a messenger ribonucleoprotein pull-down assay. Overexpression of the CCDC152 gene affected neither the expression levels nor the localization of SBP2 protein in HepG2 cells (Supplementary Figure S4D and E). In the pull-down experiment using whole-cell lysates of *CCDC152*-overexpressing HepG2 cells and a biotinylated SELENOP mRNA, a decrease in the binding of the SBP2 protein to the SELENOP mRNA was observed in *CCDC152*-overexpressing HepG2 cells (Figure 5C). Furthermore, the interaction between SBP2 and the SELENOP mRNA in the nuclear was evaluated via SBP2 immunoprecipitation of the nuclear fraction (Supplementary Figure S4F). The immunoprecipitants of the nuclear fraction were subjected to the quantitative PCR analysis of SELENOP mRNA levels, and GPx4 mRNA levels were also determined as a control. The quantitative PCR analysis of SBP2 immunoprecipitants indicated the specific and significant decrease in SBP2-bound SELENOP mRNA levels, but not in SBP2-bound GPx4 mRNA levels in *CCDC152*-overexpressing HepG2 cells (Figure 5D). Further, in the pull-down experiment using a nuclear fraction of *CCDC152*-overexpressing HepG2 cells and a biotinylated SELENOP mRNA, a decrease in the binding of the SBP2 protein to the SELENOP mRNA was observed in *CCDC152*-overexpressing HepG2 cells (Supplementary Figure S4G). These results suggest that the CCDC152 gene downregulates SELENOP via the inhibition of the binding of the SELENOP mRNA to the SBP2 protein in the nuclear. Based on these properties of the CCDC152 gene, we called its product a lncRNA inhibitor of SELENOP translation (L-IST).

### EGCg treatment up-regulates *L-IST* and downregulates SELENOP

**Figure 6.**
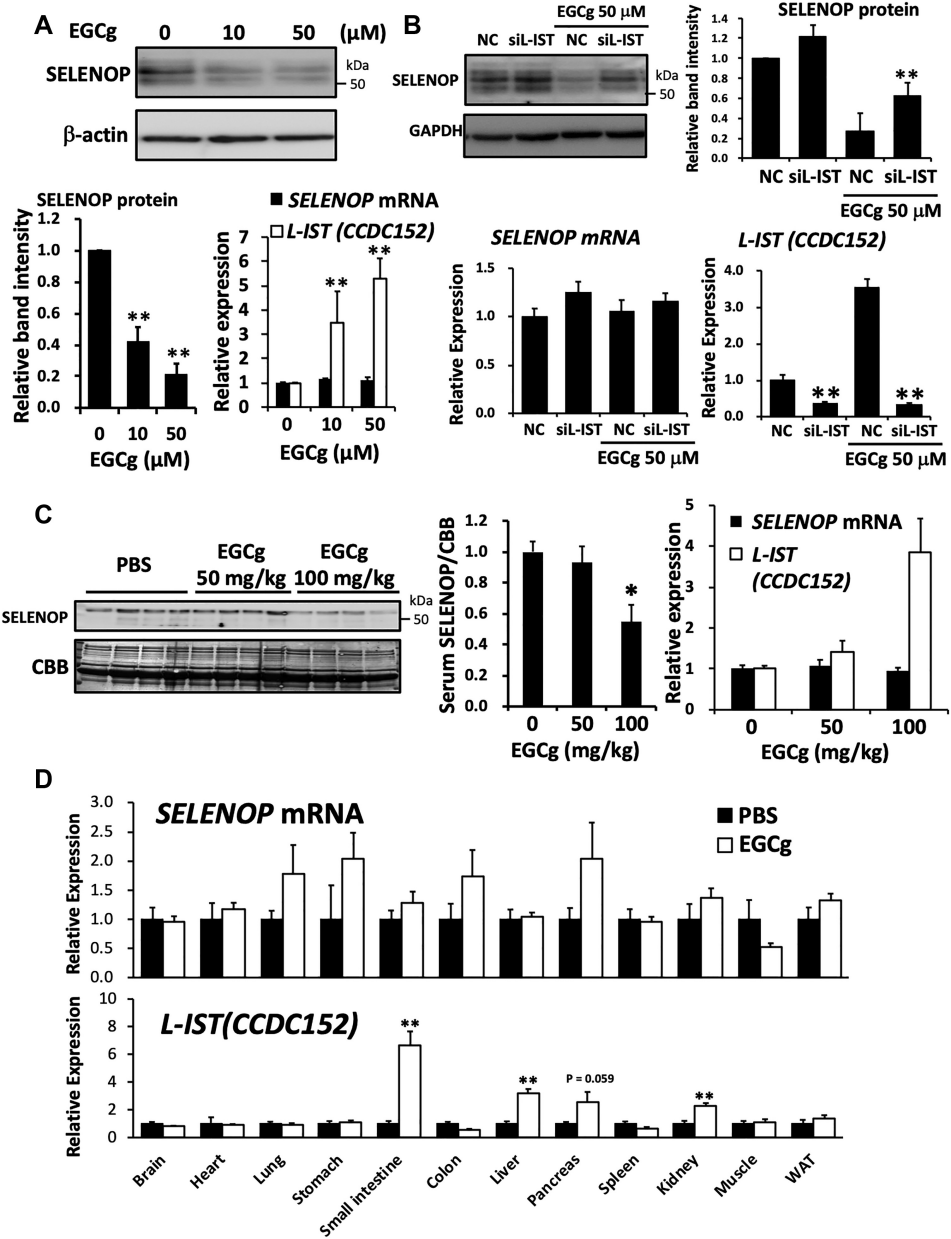
EGCg treatment up-regulated *L-IST* and downregulated SELENOP. (**A**) Effects of EGCg on *L-IST* and SELENOP expression in HepG2 cells. HepG2 cells were treated with EGCg for 24 h, and then the total RNA and whole-cell lysates were subjected to real-time PCR analysis for the SELENOP mRNA and *L-IST (CCDC152)*, followed by western blotting for SELENOP protein (*n* = 4, mean ± SD). Each of the RNA levels were normalized to that of the RPL32 mRNA, and relative expression levels to control condition (0 μM) are shown. **P < 0.01, ANOVA, Dunnett. (**B**) Effects of *L-IST*-siRNA on SELENOP protein levels in EGCg-treated HepG2 cells. HepG2 cells were treated with *L-IST (CCDC152)*-siRNA (siL-IST) or non-specific RNA (negative control, NC), and then the cells were treated with EGCg for 24 h. Total RNA and whole-cell lysates were subjected to real-time PCR analysis for the SELENOP mRNA and *L-IST*, followed by western blotting for SELENOP protein (*n* = 4, mean ± SD). **P < 0.01 versus NC, Tukey-ANOVA. (**C**, **D**) Effects of EGCg on *L-IST* and SELENOP expression in C57BL/6J mice. C57BL/6J mice were injected with EGCg (50 and 100 mg/kg intraperitoneally) or vehicle control (PBS intraperitoneally) 24 h before sampling. After serum samples were collected and perfusion with saline, total RNA samples of liver tissues were analysed by real time PCR (C, *n* = 4, mean ± SEM). SELENOP mRNA and *L-IST (CCDC152)* levels were normalized to that of the RPL32 mRNA, and relative expression levels to control condition (0 mg/kg) are shown. Serum samples were subjected to western blotting with an anti-mSELENOP Ab. CBB staining was used as a control for protein loading. Graphs display the results of densitometric quantification, normalized to the major protein (albumin, indicated by the black arrowhead) stained with CBB (C, *n* = 4, mean ± SEM). *P < 0.05, ANOVA, Dunnett. RNA isolated from each mouse tissue from animals that were injected with EGCg (100 mg/kg intraperitoneally) was subjected to real-time PCR analysis (D, *n* = 4–5, mean ± SEM). SELENOP mRNA and *L-IST (CCDC152)* levels were normalized to that of the RPL32 mRNA, and relative expression levels to PBS treatment are shown. **P < 0.01 versus PBS, Student's *t* test.

We examined several antioxidants and polyphenols to identify compounds that up-regulate *L-IST* and identified epigallocatechin gallate (EGCg) as an inducer of this molecule. Other antioxidants such as α-tocopherol and resveratrol did not change SELENOP and *L-IST* levels (data not shown). EGCg is a polyphenol that is contained in green tea and has anti-diabetic effects. As shown in Figure 6A, EGCg up-regulated *L-IST* in a concentration-dependent manner, but did not change the expression levels of the SELENOP mRNA in HepG2 cells. In EGCg-treated HepG2 cells, SELENOP levels were significantly reduced (Figure 6A). To further evaluate the role of *L-IST* elevation in EGCg-induced SELENOP decrease, we examined the effects of *L-IST*-siRNA. As shown in Figure 6B, *L-IST*-siRNA significantly decreased both endogenous *L-IST* and EGCg-induced *L-IST* elevation, while SELENOP mRNA was not changed. Further, *L-IST*-siRNA resulted in the inhibitory effects on the decrease of SELENOP protein induced by EGCg treatment (Figure 6B). These results suggested the role of L-IST in EGCg-induced SELENOP reduction. The administration of EGCg to C57BL/6J mice resulted in the increase of *L-IST* in the liver, but not in the SELENOP mRNA (Figure 6C). EGCg treatment significantly decreased the blood concentration of SELENOP (Figure 6C). We further determined the levels of *L-IST* and SELENOP mRNA in several tissues. We found that EGCG treatment significantly increased *L-IST* levels not only in the liver, but also the small intestine and kidney (Figure 6D). In contrast, SELENOP mRNA levels did not change significantly (Figure 6D).

## DISCUSSION

**Figure 7.**
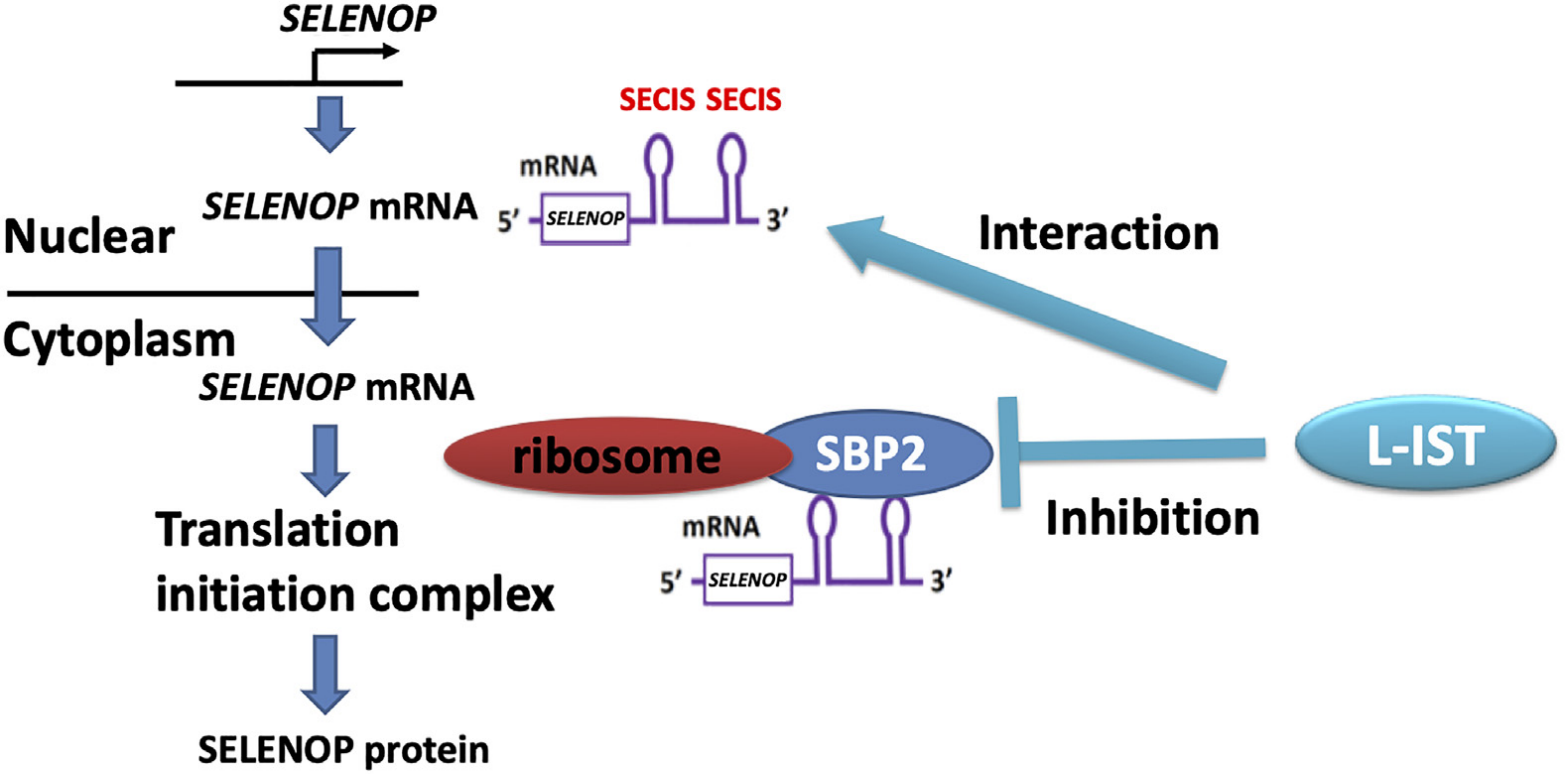
Schematic representation of the molecular mechanisms to decrease SELENOP protein expression by *L-IST*. *L-IST* directly interacts with SELENOP mRNA, inhibits the binding of SECIS-binding protein 2 (SBP2) and ribosome to SELENOP mRNA, and finally decreases SELENOP protein expression.

SBP2 and Sec-tRNA^[Ser]Sec^ play critical roles in the synthesis of selenoproteins, and defects and/or dysfunctional mutation in these factors decrease selenoprotein levels, resulting in multiple disorders (6,41). In this report, we identified *L-IST* as a novel regulator of SELENOP via the modification of the interaction between SBP2 and the SELENOP mRNA, suggesting a specific mechanism for the translational control of SELENOP (Figure 7).

A recent study has reported that the up-regulation of SELENOP is related to an increase in insulin resistance and exercise resistance, a decrease in insulin secretion and the proliferation of smooth muscle cells in pulmonary hypertension (42–45). A SELENOP-neutralizing antibody that inhibits SELENOP uptake and Se transport might be an effective therapy for diseases related to SELENOP excess (43); however, this would not be a radical treatment, as this method cannot suppress SELENOP production or reduce SELENOP concentration. It has been shown that therapeutic drugs used for diabetes, such as metformin and exendin-4, can suppress SELENOP expression in the liver (46,47); however, the targets of these reagents include a wide range of genes and proteins and their specificity for SELENOP is low. Thus, the high specificity of *L-IST* for SELENOP translation is a merit of this strategy. EGCg, a major catechin component of green tea, prevents and treats type 2 diabetes in humans and rodents. Onishi *et al.* reported recently that green tea extracts reduce SELENOP levels in the liver, although the underlying mechanism is unclear (48). The present study showed that EGCg increased *L-IST* expression in the liver and suppressed SELENOP levels in the blood. The anti-diabetic function of EGCg has been reported, and the relation between favorable action of EGCg on glucose metabolism and the effects on both *L-IST* expression and SELENOP levels is a significant subject of future study. EGCg might be a good candidate lead compound for the development of effective medicines for patients with diabetes who have high SELENOP levels.

Various mechanisms have been proposed to explain the translational control afforded by ncRNAs. Antisense UCHL1, an antisense RNA of the UCHL1 mRNA, promotes translation by recruiting the ribosome to the UCHL1 mRNA (30,49). Antisense Base1 inhibits the degradation of the target Base-1 mRNA and increases the levels of the BASE-1 protein (50). It has been reported that these antisense ncRNAs exhibit biological function via direct binding to target mRNAs. ceRNA, which do not bind directly to the target mRNA, promote its translation by trapping its miRNA (31,51). *L-IST* binds directly to its target mRNA; thus, it is considered to belong to the former ncRNA group with high specificity. *L-IST* bound to the SELENOP mRNA but did not bind to other mRNAs containing a SECIS element, such as GPx4 and TrxR1. siRNAs are ncRNAs that bind directly to their target mRNA, induce the degradation of the mRNA by *AGO1* and downregulate its protein product (52). Conversely, *L-IST* did not show any action that promoted the decomposition of its target mRNA, suggesting that *L-IST* is different from known ncRNAs because it binds to its target mRNA directly. It has been reported that RNA sequences other than antisense are necessary for translational control of ncRNAs (30). For example, antisense UCHL1 requires the SINE B2 sequence for its biological function, which exists outside of the overlapping region (30,49). The translational control of SELENOP by *L-IST* also required another RNA sequence other than the overlapping region. It is possible that new regulatory mechanisms will be found by advancing the domain analysis of the L-IST RNA. It is hypothesized that *L-IST* induces conformational changes by interacting with the SELENOP mRNA, which might result in the inhibition of the interaction between the SECIS element and the SBP2 protein. It also needs attention that the regulatory role of *L-IST* on the secretory pathway of SELENOP is not revealed in the present study since the evaluation of conditioned medium was not conducted throughout this study.

RNA and gene therapy have received much attention as a new therapeutic strategy that is different from that afforded by small compounds and antibodies. In the past decades, a gene-delivery system using a virus has been developed to express the telomerase gene in cancer cells driven by the human telomerase (hTERT) promoter and to kill cancer cells (53,54). In recent years, the drug-delivery system technology has been advancing and techniques for carrying RNA to specific tissues have been established. Among them, the usage of N-acetylgalactosamine (GALNAC) is a promising technique to deliver RNA to the liver specifically, for clinical applications (55). It is notable that the administration of GALNAC together with *L-IST* might be an effective therapeutic approach for patients with diabetes.

SECIS elements are *cis*-acting hairpin loop structures located in the 3′UTR of eukaryotic and archaeal selenoprotein mRNAs. However, there are differences in sequence and structure between eukaryotic and archaeal SECIS elements, and only the hairpin structure and a conserved AAA/G (AAR) motif in a stem-loop structure are common to all eukaryotic SECIS elements (1,56). It is thought that sequence-dependent conformation is important for the function of these SECIS elements. Usually, abnormal mRNAs, such as those with premature termination codons (PTCs), are degraded by the nonsense-mediated mRNA decay (NMD) system; in the absence of Se and Sec-tRNA^[Ser]Sec^, selenoprotein mRNAs are expected to be a target of the NMD system (57,58). It was postulated that the structural changes in the SELENOP mRNA induced by *L-IST* might generate PTCs; however, *L-IST* did not change the SELENOP mRNA levels and did not induce mRNA decay, suggesting that the SELENOP mRNA or *L-IST* might avoid NMD. It is unclear why the SELENOP mRNA might avoid NMD in the presence of *L-IST*, which decreases the interaction with SBP2. The avoidance of the NMD system of the SELENOP mRNA is observed in SBP2 KO mice (59). In addition, the interaction of the SELENOP mRNA with RIG-I recently reported (60). These observations suggest the existence of a complex regulatory system related to the SELENOP mRNA with an unknown mechanism that needs further elucidation.

It is notable that the CCDC152 gene was most expressed in the testis (Figure 1C), suggesting the function of this gene other than regulating SELENOP. It might be interesting to investigate possible activators to induce CCDC152 expression and its biological role, possibly as a protein, in the testis. In conclusion, we discovered a novel lncRNA termed *L-IST* that contained an antisense SECIS sequence of the SELENOP mRNA. We also found that EGCg up-regulated *L-IST* and downregulated SELENOP protein. Translational control of SELENOP by *L-IST* may be a new therapeutic strategy for SELENOP-related diseases such as diabetes and pulmonary hypertension.

## Supplementary Material

gkab498_Supplemental_File

## References

[1] Labunskyy V.M. , Hatfield D.L., Gladyshev V.N. Selenoproteins: molecular pathways and physiological roles. Physiol. Rev. 2014; 94:739–777. 24987004 10.1152/physrev.00039.2013 PMC4101630

[2] Flohe L. The labour pains of biochemical selenology: the history of selenoprotein biosynthesis. Biochim. Biophys. Acta. 2009; 1790:1389–1403. 19358874 10.1016/j.bbagen.2009.03.031

[3] Dagnell M. , Schmidt E.E., Arner E.S.J. The A to Z of modulated cell patterning by mammalian thioredoxin reductases. Free Rad. Biol. Med. 2018; 115:484–496. 29278740 10.1016/j.freeradbiomed.2017.12.029 PMC5771652

[4] Dumitrescu A.M. , Di Cosmo C., Liao X.H., Weiss R.E., Refetoff S. The syndrome of inherited partial SBP2 deficiency in humans. Antioxid. Redox. Signal. 2010; 12:905–920. 19769464 10.1089/ars.2009.2892 PMC2864657

[5] Rayman M.P. Selenium and human health. Lancet. 2012; 379:1256–1268. 22381456 10.1016/S0140-6736(11)61452-9

[6] Schoenmakers E. , Agostini M., Mitchell C., Schoenmakers N., Papp L., Rajanayagam O., Padidela R., Ceron-Gutierrez L., Doffinger R., Prevosto C. et al. Mutations in the selenocysteine insertion sequence-binding protein 2 gene lead to a multisystem selenoprotein deficiency disorder in humans. J. Clin. Invest. 2010; 120:4220–4235. 21084748 10.1172/JCI43653 PMC2993594

[7] Saito Y. , Yoshida Y. Niki E. Chemical reactivity and cellular uptake of tocopherols and tocotrienols. Vitamin E: Chemistry and Nutritional Benefits. 2019; The Royal Society of Chemistry 51–63.

[8] Lee B.J. , Worland P.J., Davis J.N., Stadtman T.C., Hatfield D.L. Identification of a selenocysteyl-tRNA(Ser) in mammalian cells that recognizes the nonsense codon, UGA. J. Biol. Chem. 1989; 264:9724–9727. 2498338

[9] Copeland P.R. , Fletcher J.E., Carlson B.A., Hatfield D.L., Driscoll D.M. A novel RNA binding protein, SBP2, is required for the translation of mammalian selenoprotein mRNAs. EMBO J. 2000; 19:306–314. 10637234 10.1093/emboj/19.2.306 PMC305564

[10] Berry M.J. , Banu L., Chen Y.Y., Mandel S.J., Kieffer J.D., Harney J.W., Larsen P.R. Recognition of UGA as a selenocysteine codon in type I deiodinase requires sequences in the 3′ untranslated region. Nature. 1991; 353:273–276. 1832744 10.1038/353273a0

[11] Walczak R. , Westhof E., Carbon P., Krol A. A novel RNA structural motif in the selenocysteine insertion element of eukaryotic selenoprotein mRNAs. RNA. 1996; 2:367–379. 8634917 PMC1369379

[12] Seale L.A. Selenocysteine beta-lyase: biochemistry, regulation and physiological role of the selenocysteine decomposition enzyme. Antioxidants. 2019; 8:357. 10.3390/antiox8090357 PMC6770646 31480609

[13] Caban K. , Kinzy S.A., Copeland P.R. The L7Ae RNA binding motif is a multifunctional domain required for the ribosome-dependent Sec incorporation activity of Sec insertion sequence binding protein 2. Mol. Cell. Biol. 2007; 27:6350–6360. 17636016 10.1128/MCB.00632-07 PMC2099609

[14] Hatfield D.L. , Tsuji P.A., Carlson B.A., Gladyshev V.N. Selenium and selenocysteine: roles in cancer, health, and development. Trends Biochem. Sci. 2014; 39:112–120. 24485058 10.1016/j.tibs.2013.12.007 PMC3943681

[15] Saito Y. , Takahashi K. Liu J. Selenoprotein P. Selenoproteins Mimics. 2012; Springer-Verlag Berlin Heidelberg 77–88.

[16] Burk R.F. , Hill K.E. Regulation of selenium metabolism and transport. Ann Rev Nutr. 2015; 35:109–134. 25974694 10.1146/annurev-nutr-071714-034250

[17] Saito Y. , Hayashi T., Tanaka A., Watanabe Y., Suzuki M., Saito E., Takahashi K. Selenoprotein P in human plasma as an extracellular phospholipid hydroperoxide glutathione peroxidase. Isolation and enzymatic characterization of human selenoprotein p. J. Biol. Chem. 1999; 274:2866–2871. 9915822 10.1074/jbc.274.5.2866

[18] Saito Y. , Takahashi K. Characterization of selenoprotein P as a selenium supply protein. Eur. J. Biochem. 2002; 269:5746–5751. 12423375 10.1046/j.1432-1033.2002.03298.x

[19] Saito Y. , Sato N., Hirashima M., Takebe G., Nagasawa S., Takahashi K. Domain structure of bi-functional selenoprotein P. Biochem. J. 2004; 381:841–846. 15117283 10.1042/BJ20040328 PMC1133894

[20] Hill K.E. , Lloyd R.S., Yang J.G., Read R., Burk R.F. The cDNA for rat selenoprotein P contains 10 TGA codons in the open reading frame. J. Biol. Chem. 1991; 266:10050–10053. 2037562

[21] Hill K.E. , Lloyd R.S., Burk R.F. Conserved nucleotide sequences in the open reading frame and 3′ untranslated region of selenoprotein P mRNA. Proc. Nat. Acad. Sci. U.S.A. 1993; 90:537–541. 10.1073/pnas.90.2.537 PMC45698 8421687

[22] Shetty S. , Copeland P.R. Molecular mechanism of selenoprotein P synthesis. Biochim. Biophys. Acta. 2018; 1862:2506–2510. 10.1016/j.bbagen.2018.04.011 PMC6188828 29656121

[23] Visel A. , Rubin E.M., Pennacchio L.A. Genomic views of distant-acting enhancers. Nature. 2009; 461:199–205. 19741700 10.1038/nature08451 PMC2923221

[24] Consortium E.P. An integrated encyclopedia of DNA elements in the human genome. Nature. 2012; 489:57–74. 22955616 10.1038/nature11247 PMC3439153

[25] Palazzo A.F. , Lee E.S. Sequence determinants for nuclear retention and cytoplasmic export of mRNAs and lncRNAs. Front. Genet. 2018; 9:440. 30386371 10.3389/fgene.2018.00440 PMC6199362

[26] Sun Q. , Hao Q., Prasanth K.V. Nuclear long noncoding RNAs: key regulators of gene expression. Trends Genet. 2018; 34:142–157. 29249332 10.1016/j.tig.2017.11.005 PMC6002860

[27] Uszczynska-Ratajczak B. , Lagarde J., Frankish A., Guigo R., Johnson R. Towards a complete map of the human long non-coding RNA transcriptome. Nat. Rev. Genet. 2018; 19:535–548. 29795125 10.1038/s41576-018-0017-y PMC6451964

[28] Carthew R.W. , Sontheimer E.J. Origins and mechanisms of miRNAs and siRNAs. Cell. 2009; 136:642–655. 19239886 10.1016/j.cell.2009.01.035 PMC2675692

[29] Yoon J.H. , Abdelmohsen K., Srikantan S., Yang X., Martindale J.L., De S., Huarte M., Zhan M., Becker K.G., Gorospe M. LincRNA-p21 suppresses target mRNA translation. Mol. Cell. 2012; 47:648–655. 22841487 10.1016/j.molcel.2012.06.027 PMC3509343

[30] Carrieri C. , Cimatti L., Biagioli M., Beugnet A., Zucchelli S., Fedele S., Pesce E., Ferrer I., Collavin L., Santoro C. et al. Long non-coding antisense RNA controls Uchl1 translation through an embedded SINEB2 repeat. Nature. 2012; 491:454–457. 23064229 10.1038/nature11508

[31] Cesana M. , Cacchiarelli D., Legnini I., Santini T., Sthandier O., Chinappi M., Tramontano A., Bozzoni I. A long noncoding RNA controls muscle differentiation by functioning as a competing endogenous RNA. Cell. 2011; 147:358–369. 22000014 10.1016/j.cell.2011.09.028 PMC3234495

[32] Tay Y. , Kats L., Salmena L., Weiss D., Tan S.M., Ala U., Karreth F., Poliseno L., Provero P., Di Cunto F. et al. Coding-independent regulation of the tumor suppressor PTEN by competing endogenous mRNAs. Cell. 2011; 147:344–357. 22000013 10.1016/j.cell.2011.09.029 PMC3235920

[33] Saito Y. , Watanabe Y., Saito E., Honjoh T., Takahashi K. Production and application of monoclonal antibodies to human selenoprotein P. J Health Sci. 2001; 47:346–352.

[34] Yarimizu J. , Nakamura H., Yodoi J., Takahashi K. Efficiency of selenocysteine incorporation in human thioredoxin reductase. Antiox Redox Signal. 2000; 2:643–651. 10.1089/ars.2000.2.4-643 11213469

[35] Engreitz J.M. , Pandya-Jones A., McDonel P., Shishkin A., Sirokman K., Surka C., Kadri S., Xing J., Goren A., Lander E.S. et al. The Xist lncRNA exploits three-dimensional genome architecture to spread across the X chromosome. Science. 2013; 341:1237973. 23828888 10.1126/science.1237973 PMC3778663

[36] Engreitz J.M. , Sirokman K., McDonel P., Shishkin A.A., Surka C., Russell P., Grossman S.R., Chow A.Y., Guttman M., Lander E.S. RNA-RNA interactions enable specific targeting of noncoding RNAs to nascent Pre-mRNAs and chromatin sites. Cell. 2014; 159:188–199. 25259926 10.1016/j.cell.2014.08.018 PMC4177037

[37] Nakagawa T. , Hattori S., Nobuta R., Kimura R., Nakagawa M., Matsumoto M., Nagasawa Y., Funayama R., Miyakawa T., Inada T. et al. The autism-related protein SETD5 controls neural cell proliferation through epigenetic regulation of rDNA expression. iScience. 2020; 23:101030. 32299058 10.1016/j.isci.2020.101030 PMC7160574

[38] Howard A.S. , Morozova N., Stoytcheva Z., Forry E.P., Mansell J.P., Harney J.W., Carlson B.A., Xu X., Hatfield D.L., Berry M.L. Supramolecular complexes mediate selenocysteine incorporation in vivo. Mol. Cell. Biol. 2006; 26:2337–2346. 16508009 10.1128/MCB.26.6.2337-2346.2006 PMC1430297

[39] Low S.C. , Grundner-Culemann E., Harney J.W., Berry M.J. SECIS-SBP2 interactions dictate selenocysteine incorporation efficiency and selenoprotein hierarchy. EMBO J. 2000; 19:6882–6890. 11118223 10.1093/emboj/19.24.6882 PMC305907

[40] Shetty S.P. , Sturts R., Vetick M., Copeland P.R. Processive incorporation of multiple selenocysteine residues is driven by a novel feature of the selenocysteine insertion sequence. J. Biol. Chem. 2018; 293:19377–19386. 30323062 10.1074/jbc.RA118.005211 PMC6302164

[41] Saito Y. , Shichiri M., Hamajima T., Ishida N., Mita Y., Nakao S., Hagihara Y., Yoshida Y., Takahashi K., Niki E. et al. Enhancement of lipid peroxidation and its amelioration by vitamin E in a subject with mutations in the SBP2 gene. J. Lipid Res. 2015; 56:2172–2182. 26411970 10.1194/jlr.M059105 PMC4617404

[42] Misu H. , Takamura T., Takayama H., Hayashi H., Matsuzawa-Nagata N., Kurita S., Ishikura K., Ando H., Takeshita Y., Ota T. et al. A liver-derived secretory protein, selenoprotein P, causes insulin resistance. Cell Metab. 2010; 12:483–495. 21035759 10.1016/j.cmet.2010.09.015

[43] Mita Y. , Nakayama K., Inari S., Nishito Y., Yoshioka Y., Sakai N., Sotani K., Nagamura T., Kuzuhara Y., Inagaki K. et al. Selenoprotein P-neutralizing antibodies improve insulin secretion and glucose sensitivity in type 2 diabetes mouse models. Nat. Commun. 2017; 8:1658. 29162828 10.1038/s41467-017-01863-z PMC5698464

[44] Misu H. , Takayama H., Saito Y., Mita Y., Kikuchi A., Ishii K.A., Chikamoto K., Kanamori T., Tajima N., Lan F. et al. Deficiency of the hepatokine selenoprotein P increases responsiveness to exercise in mice through upregulation of reactive oxygen species and AMP-activated protein kinase in muscle. Nat. Med. 2017; 23:508–516. 28263310 10.1038/nm.4295

[45] Kikuchi N. , Satoh K., Kurosawa R., Yaoita N., Elias-Al-Mamun M., Siddique M.A.H., Omura J., Satoh T., Nogi M., Sunamura S. et al. Selenoprotein P promotes the development of pulmonary arterial hypertension. Circulation. 2018; 138:600–623. 29636330 10.1161/CIRCULATIONAHA.117.033113

[46] Takayama H. , Misu H., Iwama H., Chikamoto K., Saito Y., Murao K., Teraguchi A., Lan F., Kikuchi A., Saito R. et al. Metformin suppresses expression of the selenoprotein P gene via an AMP-activated kinase (AMPK)/FoxO3a pathway in H4IIEC3 hepatocytes. J. Biol. Chem. 2014; 289:335–345. 24257750 10.1074/jbc.M113.479386 PMC3879556

[47] Tajima-Shirasaki N. , Ishii K.A., Takayama H., Shirasaki T., Iwama H., Chikamoto K., Saito Y., Iwasaki Y., Teraguchi A., Lan F. et al. Eicosapentaenoic acid down-regulates expression of the selenoprotein P gene by inhibiting SREBP-1c protein independently of the AMP-activated protein kinase pathway in H4IIEC3 hepatocytes. J. Biol. Chem. 2017; 292:10791–10800. 28465347 10.1074/jbc.M116.747006 PMC5491766

[48] Onishi S. , Kitazawa H., Meguro S., Tokimitsu I. Green tea extracts reduce leukocyte cell-Derived chemotaxin 2 and selenoprotein P levels in the livers of C57BL/6J mice fed a high-fat diet. Biosci. Biotechnol. Biochem. 2018; 82:1568–1575. 29848194 10.1080/09168451.2018.1480349

[49] Carrieri C. , Forrest A.R., Santoro C., Persichetti F., Carninci P., Zucchelli S., Gustincich S. Expression analysis of the long non-coding RNA antisense to Uchl1 (AS Uchl1) during dopaminergic cells' differentiation in vitro and in neurochemical models of Parkinson's disease. Front Cell Neurosci. 2015; 9:114. 25883552 10.3389/fncel.2015.00114 PMC4381646

[50] Faghihi M.A. , Modarresi F., Khalil A.M., Wood D.E., Sahagan B.G., Morgan T.E., Finch C.E., St Laurent G. 3rd, Kenny P.J., Wahlestedt C. Expression of a noncoding RNA is elevated in Alzheimer's disease and drives rapid feed-forward regulation of beta-secretase. Nat. Med. 2008; 14:723–730. 18587408 10.1038/nm1784 PMC2826895

[51] Salmena L. , Poliseno L., Tay Y., Kats L., Pandolfi P.P. A ceRNA hypothesis: the Rosetta Stone of a hidden RNA language. Cell. 2011; 146:353–358. 21802130 10.1016/j.cell.2011.07.014 PMC3235919

[52] Betancur J.G. , Yoda M., Tomari Y. miRNA-like duplexes as RNAi triggers with improved specificity. Front Genet. 2012; 3:127. 22807929 10.3389/fgene.2012.00127 PMC3395129

[53] Zhang Q. , Nie M., Sham J., Su C., Xue H., Chua D., Wang W., Cui Z., Liu Y., Liu C. et al. Effective gene-viral therapy for telomerase-positive cancers by selective replicative-competent adenovirus combining with endostatin gene. Cancer Res. 2004; 64:5390–5397. 15289347 10.1158/0008-5472.CAN-04-1229

[54] Mizukoshi E. , Kaneko S. Telomerase-targeted cancer immunotherapy. Int. J. Mol. Sci. 2019; 20:1823. 10.3390/ijms20081823 PMC6515163 31013796

[55] Springer A.D. , Dowdy S.F. GalNAc-siRNA conjugates: leading the way for delivery of RNAi therapeutics. Nucleic Acid Ther. 2018; 28:109–118. 29792572 10.1089/nat.2018.0736 PMC5994659

[56] Caban K. , Copeland P.R. Selenocysteine insertion sequence (SECIS)-binding protein 2 alters conformational dynamics of residues involved in tRNA accommodation in 80 S ribosomes. J. Biol. Chem. 2012; 287:10664–10673. 22308032 10.1074/jbc.M111.320929 PMC3323001

[57] He F. , Jacobson A. Nonsense-mediated mRNA decay: degradation of defective transcripts is only part of the story. Annu. Rev. Genet. 2015; 49:339–366. 26436458 10.1146/annurev-genet-112414-054639 PMC4837945

[58] Kurosaki T. , Maquat L.E. Nonsense-mediated mRNA decay in humans at a glance. J. Cell Sci. 2016; 129:461–467. 26787741 10.1242/jcs.181008 PMC4760306

[59] Fradejas-Villar N. , Seeher S., Anderson C.B., Doengi M., Carlson B.A., Hatfield D.L., Schweizer U., Howard M.T. The RNA-binding protein Secisbp2 differentially modulates UGA codon reassignment and RNA decay. Nucleic. Acids. Res. 2017; 45:4094–4107. 27956496 10.1093/nar/gkw1255 PMC5397149

[60] Murai K. , Honda M., Shirasaki T., Shimakami T., Omura H., Misu H., Kita Y., Takeshita Y., Ishii K.A., Takamura T. et al. Induction of selenoprotein P mRNA during hepatitis C virus infection inhibits RIG-I-mediated antiviral immunity. Cell Host Microbe. 2019; 25:588–601. 30974086 10.1016/j.chom.2019.02.015

