# Supplementary material for: Identification of a novel endogenous long non-coding RNA that inhibits selenoprotein P translation
Source: Nucleic Acids Res. 2021 Jun 18;49(12):6893–907. doi: 10.1093/nar/gkab498 (PMC8266573; doi:10.1093/nar/gkab498)
Supplement: gkab498_Supplemental_File [file gkab498_supplemental_file.pdf]

Supplementary figure

Title: Identification of a Novel Endogenous Long Non-coding RNA that Inhibits Selenoprotein P Translation

Yuichiro Mita*, Risa Uchida, Sayuri Yasuhara, Tadashi Yokooji, Yoshino Shirakawa, Yasuomi Urano, Noriko Noguchi, and Yoshiro Saito*

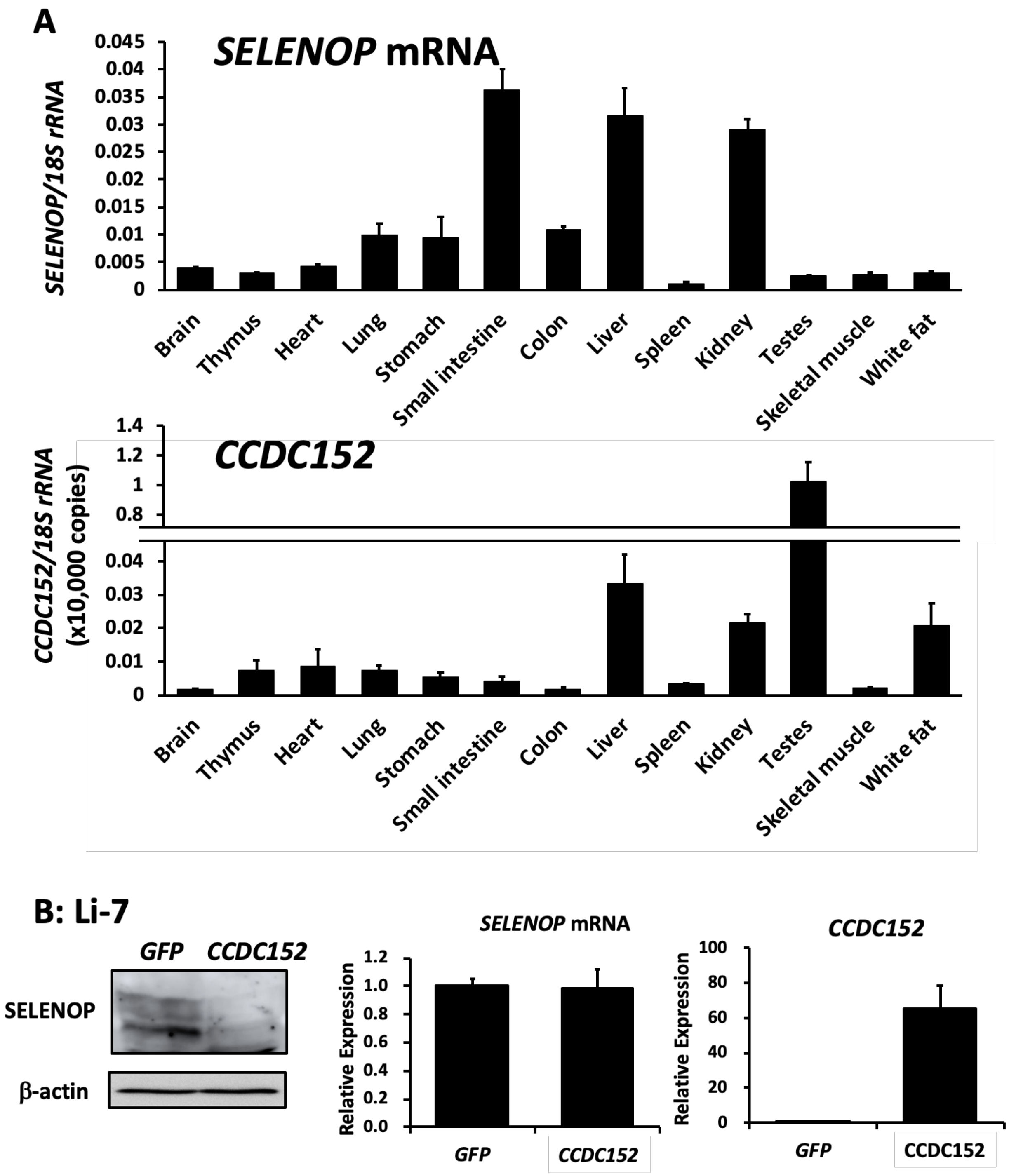

Y Mita et al, Supplementary Fig. S1

Supplementary Figure S1. A. Relative expression levels of SELENOP and the CCDC152 gene in each mouse tissue. RNA isolated from each mouse tissue was subjected to real-time PCR analysis (n = 5, mean ± SEM). **B. Effects of CCDC152 transfection on the SELENOP protein and the SELENOP and CCDC152 gene expression levels in human hepatoma Li-7 cells.** Li-7 cells were treated with CCDC152 plasmid DNA or control GFP plasmid DNA for 48 h, and then whole cell lysates and extracted total RNA were subjected to western blotting with an anti-SELENOP Ab and real-time PCR, respectively (n = 3, mean ± SD).

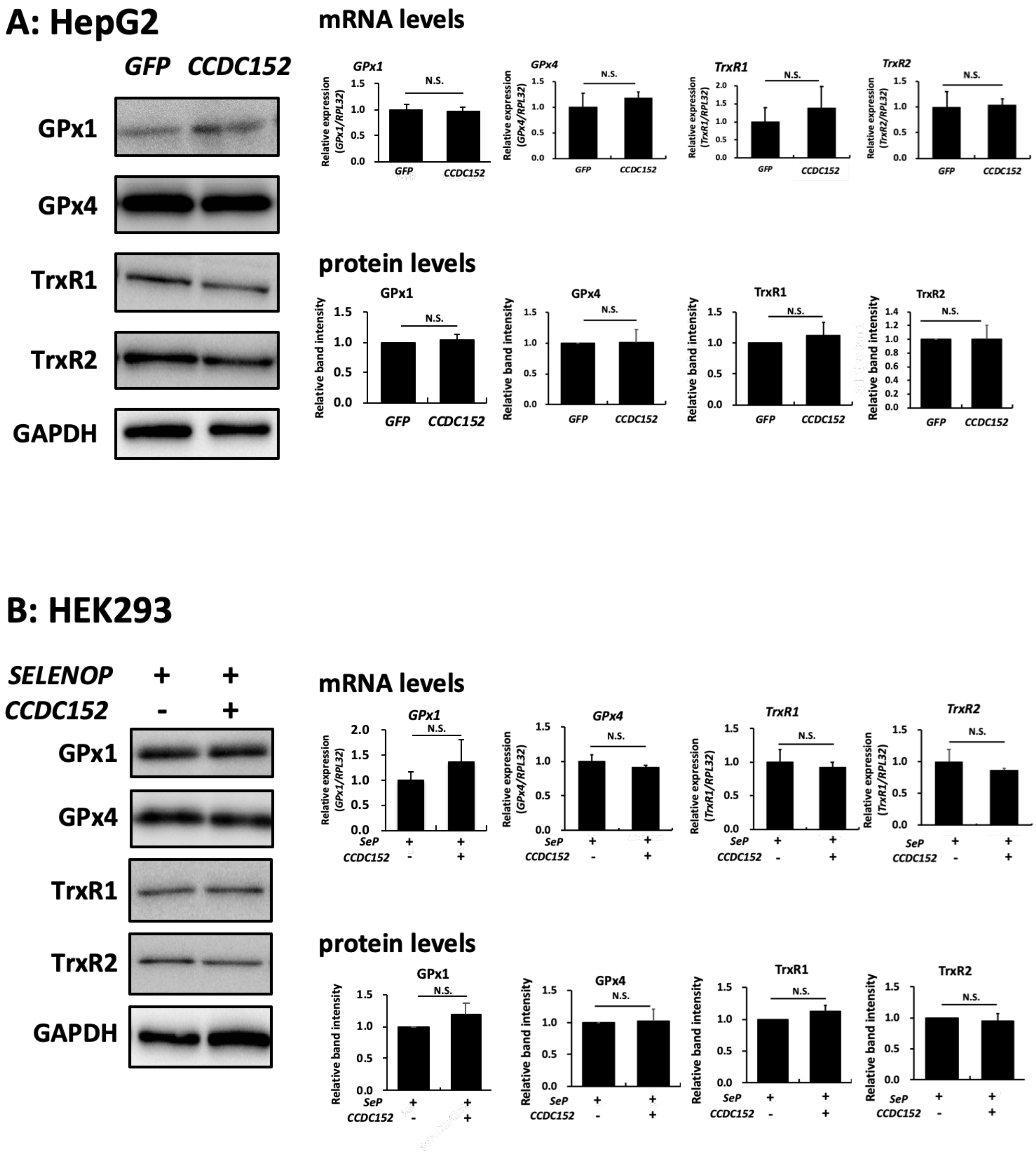

Y Mita et al, Supplementary Fig. S2

Supplementary Figure S2. A-B. Effects of CCDC152 gene expression on selenoprotein levels in HepG2 and HEK293 cells. Selenoprotein and mRNA levels in HepG2 cells (A) and HEK293 cells (B) treated with the *CCDC152* plasmid DNA or control plasmid DNA for 48 h (n = 3, mean ± SD). Extracted proteins and RNAs were subjected to western blotting with specific Ab for each selenoprotein and real-time PCR, respectively. N.S. (not significant) vs. Control, Student's *t* test.

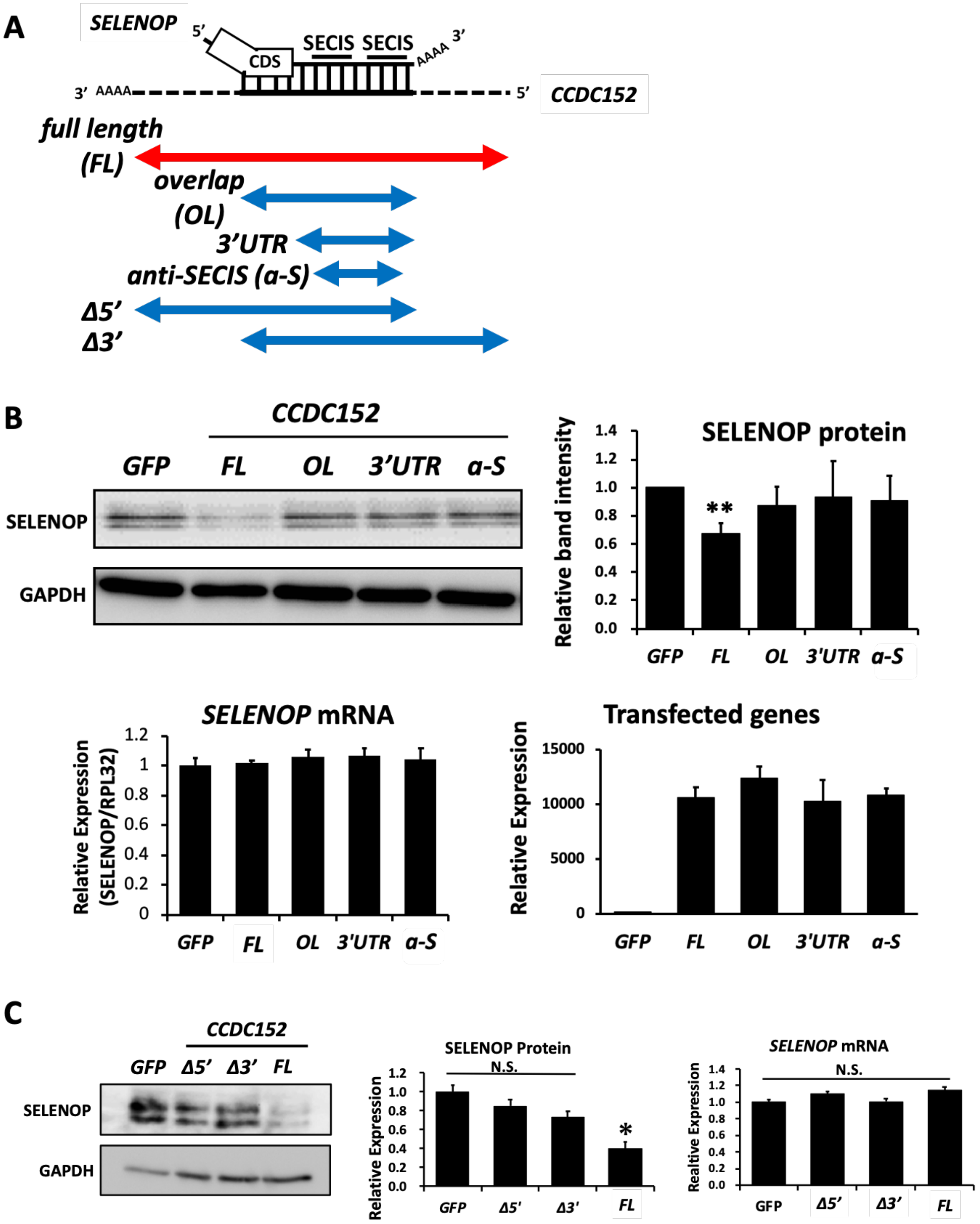

Y Mita et al, Supplementary Fig. S3

Supplementary Figure S3. A-C. A-C. Effects of CCDC152 RNA fragments on SELENOP levels of HepG2 cells. The full-length CCDC152 RNA and its fragments were synthesized in vitro and were transfected into HepG2 cells. A synthesized GFP mRNA was used as a control. After transfection, whole-cell lysates and total RNA were subjected to western blotting and real-time PCR analysis, respectively ($n = 3$, mean ± SD). $^{**}P < 0.01$, $^{*}P < 0.05$ vs. GFP control, Dunnett-ANOVA. Abbreviations: OL, overlapping region; 3′UTR, 3′UTR region; a-S, anti-SECIS region; Δ5′, deletion of the 5′ region without overlapping region; Δ3′, deletion of the 3′ region without overlapping region.

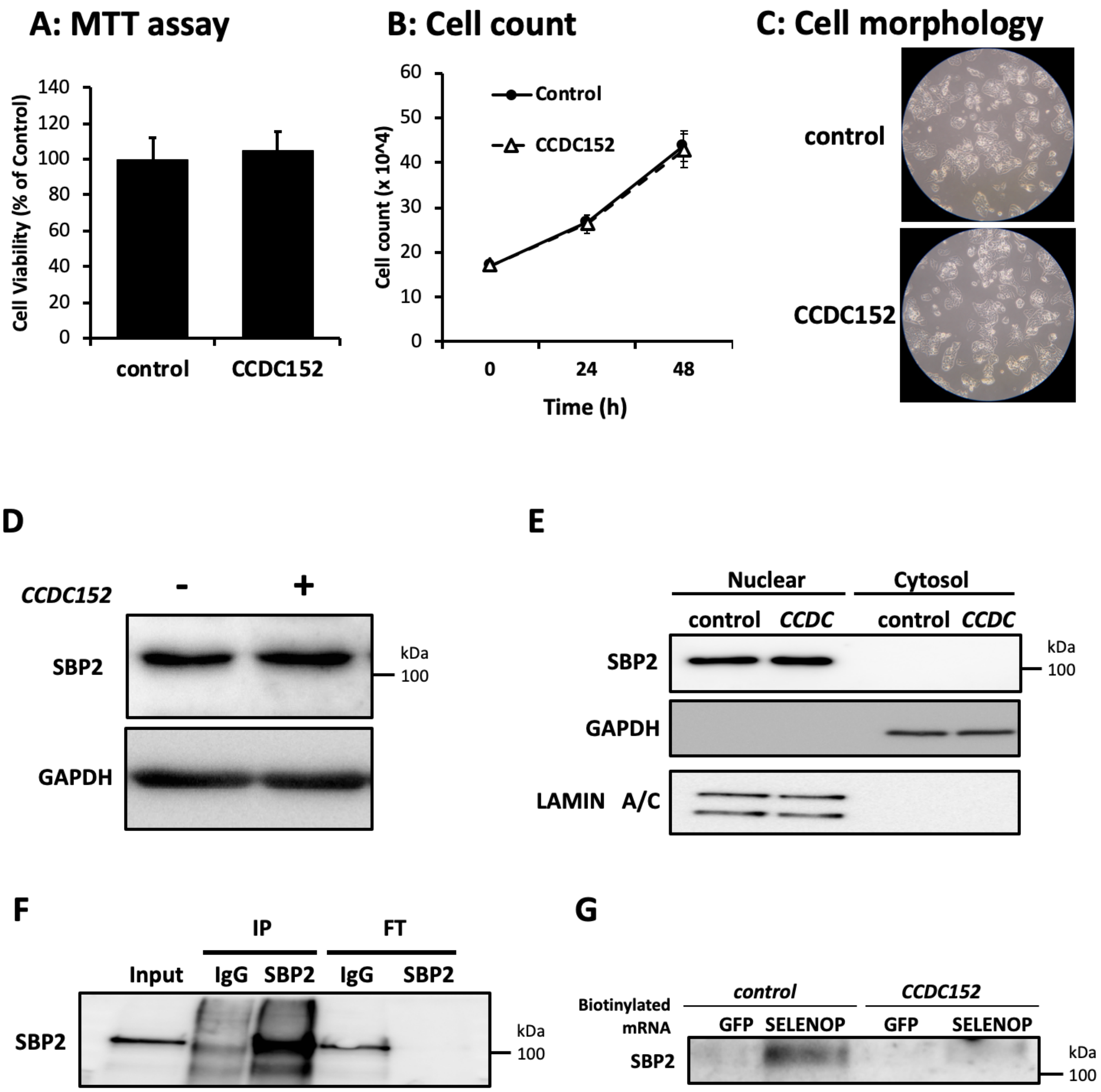

Y Mita et al, Supplementary Fig. S4

Supplementary Figure S4. A-C. Effects of CCDC152 overexpression on cell growth and morphology. The control and *CCDC152* plasmids were transfected into HepG2 cells and cell viability and cell number were determined by MTT assay (A, Cell counting kit-8, Dojindo) and cell count (B), respectively. MTT assay was conducted 48 hr after transfection. The cell morphology was confirmed 48 hr after transfection. **D. Evaluation of SBP2 protein levels in CCDC152-transfected HepG2 cells.** The control and *CCDC152* plasmids were transfected into HepG2 cells and whole-cell lysates were subjected to western blotting with the anti-SBP2 antibody. **E. Evaluation of SBP2 protein levels in the nuclear and cytosolic fraction of HepG2 cells.** The control and *CCDC152* plasmids were transfected into HepG2 cells, nuclear and cytosolic fractions were prepared, and each fraction was subjected to western blotting using an anti-SBP2 antibody. The separation of each fraction was also evaluated using marker proteins of each fraction. **F. Confirmation of SBP2 immunoprecipitation in the nuclear fraction of HepG2 cells.** An anti-SBP2 Ab or control IgG was added to each nuclear extract fraction and the proteins in the immunoprecipitants (IP) and flow-through (FT) fraction were subjected to western blot analysis using an anti-SBP2 Ab. **G. Effects of the CCDC152 gene on the interaction between SELENOP mRNA and SBP2 protein in the nuclear.** The control and *CCDC152* plasmids were transfected into HepG2 cells, each nuclear fraction was prepared, and biotinylated-GFP mRNA and SELENOP mRNA was added. The precipitants were subjected to western blotting using an anti-SBP2 antibody.

Supplementary Table S1. The sequences of the primers used in the present study.

human RPL32	F	CCCCTTGTGAAGCCCAAGA
	R	TGACTGGTGCCGGATGAAC
human SELENOP	F	CCCCCAGCCTGGAGCATAAG
	R	TGCACAGGTATCAGCTGGCTT
human L-IST	F	GGGGAACTAGGAGCAACAGC
	R	AGACCTCCTTTGCTTGCATT
human GPx4	F	GCGCTATGGTCCCATGGA
	R	GAGATAGCACGGCAGGTCCTT
human TR1	F	ATGGGCAATTTATTGGTCCTCAC
	R	CCCAAGTAACGTGGTCTTTCAC
human TR2	F	GTGGGCCATAGGTCGAGTC
	R	TGAGTGTCGGGGCTAGTATCTA
mouse 18S rRNA	F	AGGAATTGACGGAAGGGCACCA
	R	GTGCAGCCCCGGACATCTAAG
mouse SELENOP	F	AGCTCTGCTTGTTACAAAGCC
	R	CAGGTCTTCCAATCTGGATGC
mouse L-IST	F	GCAGCAGGCATGGATCAAAAC
	R	GAAGGTTGTTCTTTCCAGCAGT
